# Changes in Drug‐Induced Hospitalisations and Deaths During the First Year of the COVID‐19 Pandemic in Australia

**DOI:** 10.1111/dar.14088

**Published:** 2025-06-08

**Authors:** Nicola Man, Jane Akhurst, Olivia Price, Agata Chrzanowska, Rachel Sutherland, Paul M. Dietze, Raimondo Bruno, Louisa Degenhardt, Wing See Yuen, Lauren Moran, Louise Tierney, Amy Peacock

**Affiliations:** ^1^ National Drug and Alcohol Research Centre UNSW Sydney Sydney Australia; ^2^ Tobacco, Alcohol & Other Drugs Unit Australian Institute of Health and Welfare Canberra Australia; ^3^ Burnet Institute Melbourne Australia; ^4^ National Drug Research Institute Curtin University Perth and Melbourne Australia; ^5^ School of Psychological Sciences University of Tasmania Hobart Australia; ^6^ Mortality Data Centre, Health and Vital Statistics Section Australian Bureau of Statistics Canberra Australia

**Keywords:** big events, COVID‐19, hospitalisations, illicit drug, mortality, opioid, stimulant

## Abstract

**Introduction:**

We aimed to determine whether the trend in the rate of drug‐induced hospitalisations and deaths changed during the first year of the COVID‐19 pandemic in Australia.

**Methods:**

Data comprised crude monthly rates (per 1,000,000 persons) of hospitalisations and deaths directly attributable to illicit drugs, prescription medicines, or medicines available without a prescription, nationally from 2011 to 2021. Observed rates during the COVID‐19 pandemic (2020–2021) were compared with their counterfactual forecast in an ARIMA model, overall and disaggregated by sex, age and drug involved.

**Results:**

Observed rates of drug‐induced hospitalisation and death, overall and by sex, were not significantly different from the forecasted rates. The rates of drug‐induced death among people aged 35–54 and 55+ years were lower than forecasted by 2.1 [95% prediction interval = −3.8, −0.4] and 0.7 [−1.3, −0.1] deaths per 1,000,000 persons per month, respectively. The rates of drug‐induced hospitalisation and death involving heroin were lower than forecasted by 1.5 [−2.4, −0.7] and 1.0 [−1.3, −0.6] per 1,000,000 persons per month, respectively, as were those involving amphetamine‐type stimulants by 12.4 [−21.4, −0.8] and 0.5 [−0.7, −0.2] per 1,000,000 persons per month, respectively. The rate of cannabinoid‐induced hospitalisations was higher than forecasted by 3.8 [0.8, 6.8] hospitalisations per 1,000,000 persons per month.

**Discussion and Conclusions:**

We found no evidence of an overall difference in the rate of drug‐induced harms during the COVID‐19 pandemic relative to the forecasted trend. However, there were differences by drug involved, which may be explained by drug market disruptions and changes in drug use during the pandemic.

## 1 Introduction

The onset of the COVID‐19 pandemic raised concerns about increased drug‐induced harms in Australia, among other countries [1]. The introduction of public health measures (e.g., stay‐at‐home orders) amplified existing social and economic disparities and introduced new stressors (e.g., financial instability) that precipitated poor mental health and escalated drug use for some people [2, 3, 4, 5, 6]. There was an increased risk of harm (particularly overdose) as social distancing policies led to an increase in people using drugs alone [7] and supply chain disruptions resulted in changes to purity and contents of illicit drugs [8]. Moreover, drug treatment and harm reduction agencies experienced challenges (e.g., staff shortages) maintaining services such as take‐home naloxone and opioid agonist treatment [9].

Early evidence suggested there was a rise in drug‐induced deaths during the pandemic in other countries with universal healthcare systems, including Canada and England [10, 11]. In the US, excess mortality related to drug overdose accelerated during the pandemic, with greater increases observed among women, adolescents and for deaths involving fentanyl [12]. Preliminary data from Europe suggested drug‐related deaths might have been lower in 2020 compared to 2019 in some countries, including Austria and Norway, but higher in Germany and Sweden [13]. Together, these findings suggest that changes to drug‐related harm during the COVID‐19 pandemic varied depending on the population and setting.

Australia's experience of the COVID‐19 pandemic was unique in many respects, hence impacts on drug‐induced harms may have differed. Although gross domestic product declined and unemployment increased, economic measures introduced to increase the income of people receiving government benefits and provide income to those who lost their jobs reduced income inequality during 2020 [14]. During the first year of the pandemic, long‐term lockdowns were limited to Melbourne (Australia's second largest city by population). Nevertheless, the restrictions still led to lower life satisfaction and worse health and wellbeing outcomes nationally [15]. The Australian illicit drug supply was disrupted, likely as a result of closed international borders, with heroin and methamphetamine in particular perceived to be harder to find, more expensive and lower in purity [16, 17]. These disruptions, in addition to fewer opportunities to socialise, led some people to reduce their drug use [18, 19]. Qualitative interviews showed that people had difficulties adapting to service changes during the lockdowns [20] and visits to Australia's two supervised injecting sites (in Sydney and Melbourne) declined during the pandemic [21], although increased access to take‐home opioid agonist treatment doses was noted as a positive outcome [20].

To date, formal statistical analyses of drug‐induced harms in Australia during COVID‐19 have been limited to specific regions. A study of opioid‐related ambulance attendances in the state of Victoria found a substantial decrease in heroin‐related ambulance attendances during the pandemic, although there was no change in attendances for pharmaceutical opioids [22]. The authors posited that the reduction in heroin‐related attendances may have been a result of interrupted supply. In contrast, emergency department presentations related to substance use in the Western Sydney region of New South Wales increased in the first half of 2020 compared to the equivalent period in 2019, although the COVID‐19 affected study period began in January 2020, before border closures and social distancing measures [23]. These studies may not reflect the overall extent of drug‐induced harm during COVID‐19 in Australia.

Prior to the COVID‐19 pandemic in Australia, drug‐induced deaths were most common among males aged 35–54 years and opioids were the most identified substances involved [24]. Drug‐induced hospitalisations occurred at similar rates between males and females, were most common among those aged 20–39 years, and amphetamine‐type stimulants were the most common substance involved [25]. Examining whether any changes to the rate of drug‐induced harms during the pandemic differed by age, sex or drug involved is important to understand whether certain profiles of people who use drugs were disproportionately affected.

Thus, the aims of this study were to investigate:

1. Whether trends in overall rates of drug‐induced hospitalisations and deaths changed with the onset of the COVID‐19 pandemic and associated restrictions in Australia through to mid‐2021.
2. Whether the change in trends varied by sex, age group and drug involved.

## 2 Methods

### 2.1 Study Design

This was a time‐series analysis of drug‐induced hospitalisations and deaths. We adopted a counterfactual forecast approach, meaning that observed events during the pandemic (i.e., from March 2020) were compared to forecasted events based on the pre‐pandemic time series. This technique has previously been used to estimate drug overdose deaths in excess of the forecasted time series in the US [12, 26]. Although similar studies have used an interrupted time series approach [21, 22, 27], the multiple changes in policies on travel and gathering restrictions during the COVID‐19 pandemic and differences across jurisdictions made it difficult to determine the additional breakpoints for such an analysis in the Australian national context [28]. Findings are reported according to the Strengthening the Reporting of Observational Studies in Epidemiology (STROBE) reporting guideline (Appendix A in Supporting Information; [29]). We had no pre‐determined registered hypotheses; results should be considered exploratory.

### 2.2 Data Sources

*National Hospital Morbidity Database (NHMD)*. The NHMD is a collection of electronic confidentialised summary records for hospitalisations (i.e., completed episodes of care) in all public and private hospitals in Australia. Diagnoses were coded according to the International Classification of Diseases and Related Health Problems, Tenth Revision Australian Modification (ICD‐10‐AM) (see Table B1 in Supporting Information for codes). Hospitalisations with a length of stay longer than 60 days were excluded as the admission month of hospital separation was not provided (< 0.5% of drug‐induced hospitalisations). Cross‐border hospitalisations, that is, records of patients whose state of usual residence was not the state of hospitalisation, were not provided and thus were not included. Hospitalisations were extracted where there was a drug‐related principal diagnosis and age at the time of admission was ≥ 15 years.

*Cause of Death Unit Record Files (COD URF)*. The COD URF includes all deaths that occurred and were registered in Australia. Drug‐induced deaths which occurred by the end of a given month where the age of the deceased person was ≥ 15 years and registered and received by the Australian Bureau of Statistics (ABS) by the first quarter of 2022 were extracted from the COD URF. Drug‐induced deaths were identified by the presence of any drug‐related ICD‐10 codes in the underlying cause of death (see Table B2 in Supporting Information for codes). The majority of drug‐induced deaths are certified by a coroner and the ABS undertake a revision process for coroner‐certified deaths over a 3‐year period [30].

National data on deaths with date of occurrence and hospitalisations with an admission date between 1 July 2011 and 30 April 2021 were studied. The former date was the earliest date on which we have data on month of hospital admissions for all jurisdictions. Data were available for hospitalisations with a separation date on or before 30 June 2021. Therefore, April 2021 was the latest month that data for all hospital admissions within the scope of the study (i.e., ≤ 60 days) were available.

### 2.3 Measures

Our primary outcome measures were the crude monthly rates (per 1,000,000 persons) of drug‐induced hospitalisations and deaths attributed to illicit drugs, as well as some prescription medicines and medicines available without a prescription. The quarterly estimated national resident population data were used for the computation of rates (as monthly data were not available) [31]. Rates were also calculated by sex (male, female), age at date of admission or date of death (15–34, 35–54, ≥ 55 years) and drug involved. For the latter, we focused on the primary broad drug classes involved in drug‐induced hospitalisations and deaths in Australia (see Table B3 in Supporting Information for further information): (i) heroin; (ii) other opioids (comprising natural and semi‐synthetic opioids, methadone, other synthetic opioids, and other and unspecified opioids/narcotics, but excluding heroin and opium); (iii) amphetamine‐type stimulants (ATS; including methamphetamine, amphetamine, MDMA and caffeine); (iv) cocaine; and (v) cannabinoids. An ‘all opioids’ class was also analysed. Opioid‐induced hospitalisations additionally include the F11 diagnosis code, which does not differentiate heroin from other opioids, that is, the number of all opioid‐related hospitalisations is generally more than that of the sum of heroin and other opioid‐related hospitalisations (see Table B3 in Supporting Information). Drug involved was identified based on principal diagnosis for hospitalisations and up to 20 multiple causes of death for drug‐induced deaths (see Table B3 in Supporting Information for ICD‐10‐AM and ICD‐10 codes).

### 2.4 Data Analysis

Autoregressive integrated moving average (ARIMA) models were fitted to the time series data from 1 July 2011 until February 2020 for the overall rates and rates by sex, age and drug type. We chose March 2020 as the first time point for the pandemic period given the World Health Organization declaration of the COVID‐19 pandemic on 11 March 2020 and the start of nationwide restrictions on 23 March 2020. The time series analyses were performed using the *fable* package in *R* [32]. The automated algorithm in the *fable* package was used to determine the ARIMA model of best fit. Forecast of the counterfactual trend over time was then performed using the ARIMA model for each time series from March 2020 until April 2021. To account for non‐independence of data and potential non‐normality of the residuals, the residuals were bootstrapped (using the bootstrap option in the forecast function) to produce 40,000 samples from which the forecast estimates and corresponding prediction intervals (PI) was computed. The difference of the observed rates from the counterfactual estimates were averaged for each time series to obtain a pooled estimate of deviation in observed rate, with corresponding 95% PI presented in a table in the results. We also plotted the observed monthly rates and counterfactual estimates with 80% and 95% PIs produced from the bootstrap samples. The 80% and 95% PIs are the typical intervals used in time series forecasts [33]. To interpret the results, we first interpreted the pooled estimate of deviation in observed rate per month, which was deemed to be significantly different from 0 if the 95% PI did not include 0. Where the pooled estimate was significantly different, we also described how the observed monthly rates in the plots differed from the predicted estimates, that is, the time period over which the monthly rates were outside of the 80% or 95% PIs.

Model assumptions were assessed with unit root tests, histograms, and autocorrelation and partial autocorrelation plots (provided in Appendix C in Supporting Information).

### 2.5 Sensitivity Analyses

The COVID‐19 pandemic and associated restrictions impacted hospital service availability, which resulted in reduced all‐cause hospitalisation rates in Australia [34]. Therefore, we performed a sensitivity analysis adjusting for all‐cause hospitalisation rates using data from 1 July 2016 onwards (when data on all‐cause hospitalisations were available). We also presented the analysis from 1 July 2016 without adjustment for all‐cause hospitalisation rates to assess whether any differences observed were due to the adjustment and/or the use of a shorter time series.

### 2.6 Ethics

Ethics approval for the study was provided by the UNSW Human Research Ethics Committees (HREC; HC220754), the Australian Institute of Health and Welfare data custodian, Royal Melbourne Hospital HREC (HREC/101690/MH‐2023), South Australia Department of Health and Wellbeing HREC (2020/HRE0043) and jurisdictional data custodians.

## 3 Results

### 3.1 Trend in Rates of Drug‐Induced Hospitalisations and Deaths, Overall and by Sex

**FIGURE 1:**
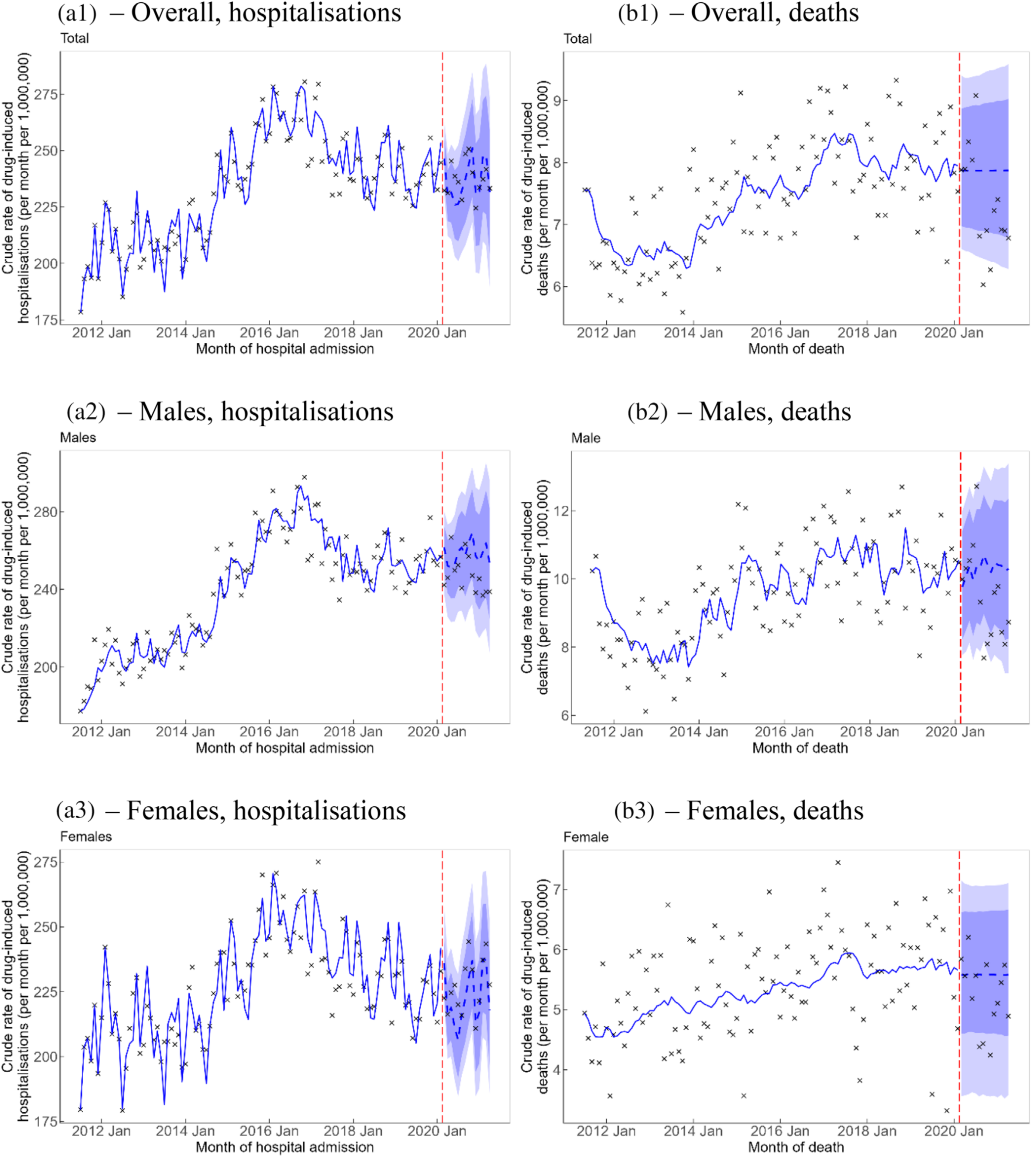
Crude rate (per month per 1,000,000 persons) of drug‐induced hospitalisations (A) and deaths (B)—overall (A1 and B1) and by sex (A2, A3, B2 and B3). Vertical red dashed line represents the onset of the COVID‐19 pandemic. Crosses represent observed rates. Blue solid line represents the estimated trend and blue dashed line represents counterfactual forecast of the trend after the onset of the COVID‐19 pandemic. Light blue and dark blue bands represent the 80% and 95% prediction intervals, respectively, of counterfactual forecast of the rates after the onset of the COVID‐19 pandemic.

The observed rates of drug‐induced hospitalisation and death, overall and by sex, were not significantly different from the predicted rates during the COVID‐19 pandemic in the counterfactual analysis, noting that the 95% PI for the rate of drug‐induced hospitalisation was relatively wide (Table 1 and Figure 1).

**TABLE 1:** Deviation in observed rate (per month per 1,000,000 persons) of drug‐induced hospitalisations and deaths from the counterfactual forecast in the COVID‐19 pandemic period.

	Drug‐induced hospitalisations	Drug‐induced deaths
Overall	0.5 (−25.1, 25.2)	−0.6 (−1.4, 0.3)
Sex		
Males	−11.0 (−35.7, 15.7)	−0.8 (−2.5, 0.9)
Females	4.5 (−16.9, 24.1)	−0.3 (−1.0, 0.4)
Age, years		
15–34	15.0 (−24.8, 57.6)	0.3 (−0.1, 0.8)
35–54	−20.3 (−48.5, 7.2)	**−2.1 (−3.8, −0.4)**
55+	−2.5 (−10.9, 5.7)	**−0.7 (−1.3, −0.1)**
Drug involved		
Opioids	**−3.3 (−6.3, −0.5)**	−0.7 (−1.5, 0.1)
Heroin	**−1.5 (−2.4, −0.7)**	**−1.0 (−1.3, −0.6)**
Other opioids	−0.7 (−2.1, 0.7)	−0.1 (−0.6, 0.6)
Amphetamine‐type stimulants	**−12.4 (−21.4, −0.8)**	**−0.5 (−0.7, −0.2)**
Cocaine	0.9 (−0.4, 1.9)	n.p.
Cannabinoids	**3.8 (0.8, 6.8)**	−0.2 (−0.5, 0.2)

*Note*: Bolded rows indicate estimates with 95% PI that does not include 0. ‘Other opioids’ exclude opium and heroin. n.p., not published because the number of drug‐induced deaths involving cocaine were too small, that is, < 5 events in at least 1 month of data. For data exclusions, please refer to the methods section and Appendix B (Tables B1 and B3 in Supporting Information).

Abbreviation: PI, prediction interval.

### 3.2 Trend by Age

**FIGURE 2:**
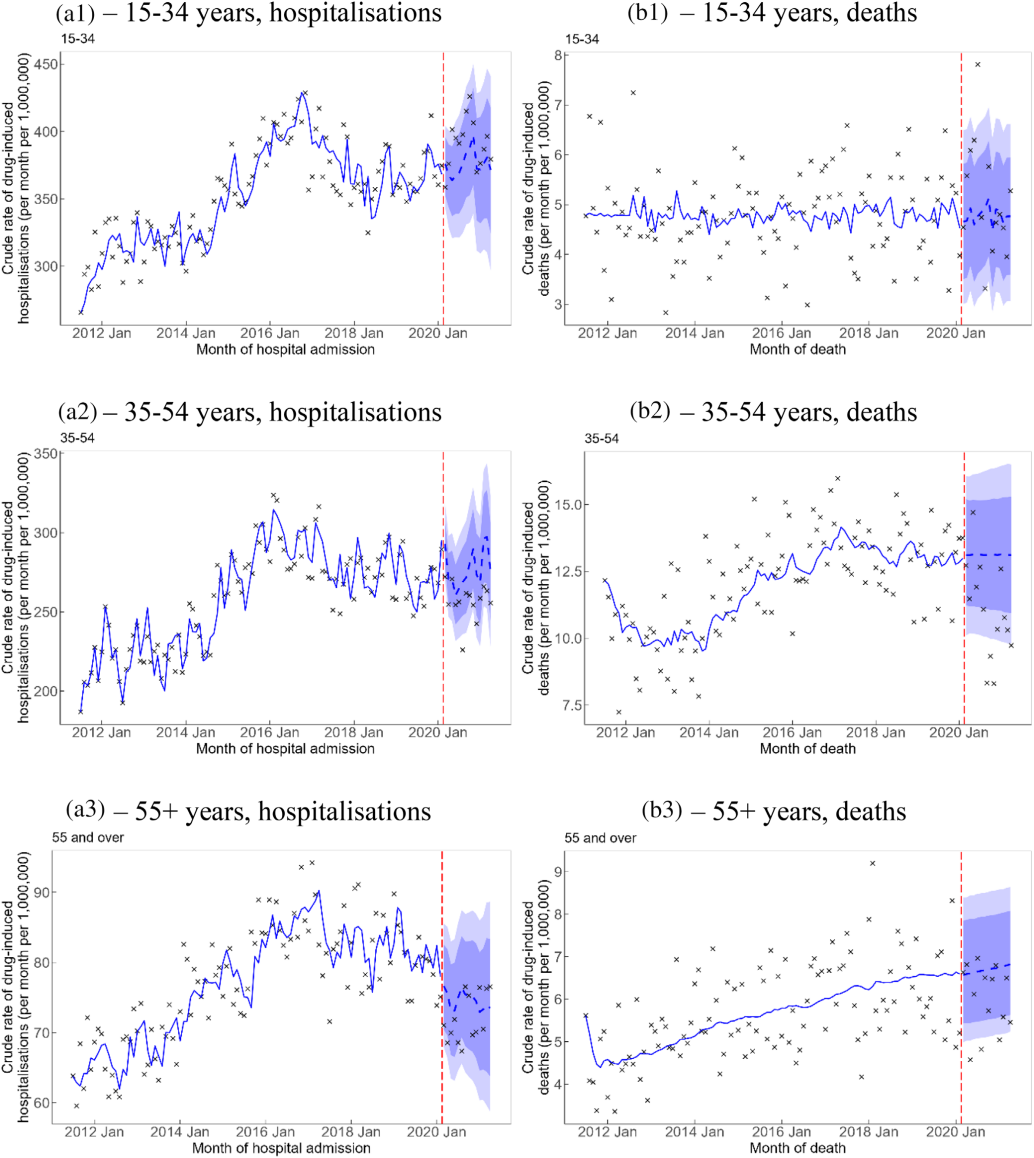
Crude rate of drug‐induced hospitalisations (A1–A3) and deaths (B1–B3) by age group. Vertical red dashed line represents the onset of the COVID‐19 pandemic. Crosses represent observed rates. Blue solid line represents the estimated trend and blue dashed line represents counterfactual forecast of the trend after the onset of the COVID‐19 pandemic. Light blue and dark blue bands represent the 80% and 95% prediction intervals, respectively, of counterfactual forecast of the rates after the onset of the COVID‐19 pandemic.

When comparing the observed with the counterfactual estimates, people aged 35–54 and 55+ years had rates of drug‐induced death that were lower than forecasted by 2.1 (95% PI −3.8, −0.4) and 0.7 (95% PI −1.3, −0.1) per 1,000,000 persons per month, respectively. The counterfactual forecast in Figure 2 shows that the observed rates of drug‐induced deaths among people aged 35–54 years was mostly below the 80% PI of the counterfactual forecast from August 2020 onwards. In contrast, the rate of drug‐induced deaths among people aged 55+ years was mostly within the 80% PI and the significance of the pooled estimate was marginal with the lower limit of the 95% PI being close to 0 (Figure 2). The rates of drug‐induced hospitalisation by age were not significantly different from the predicted rates.

### 3.3 Trend by Drug Type

**FIGURE 3:**
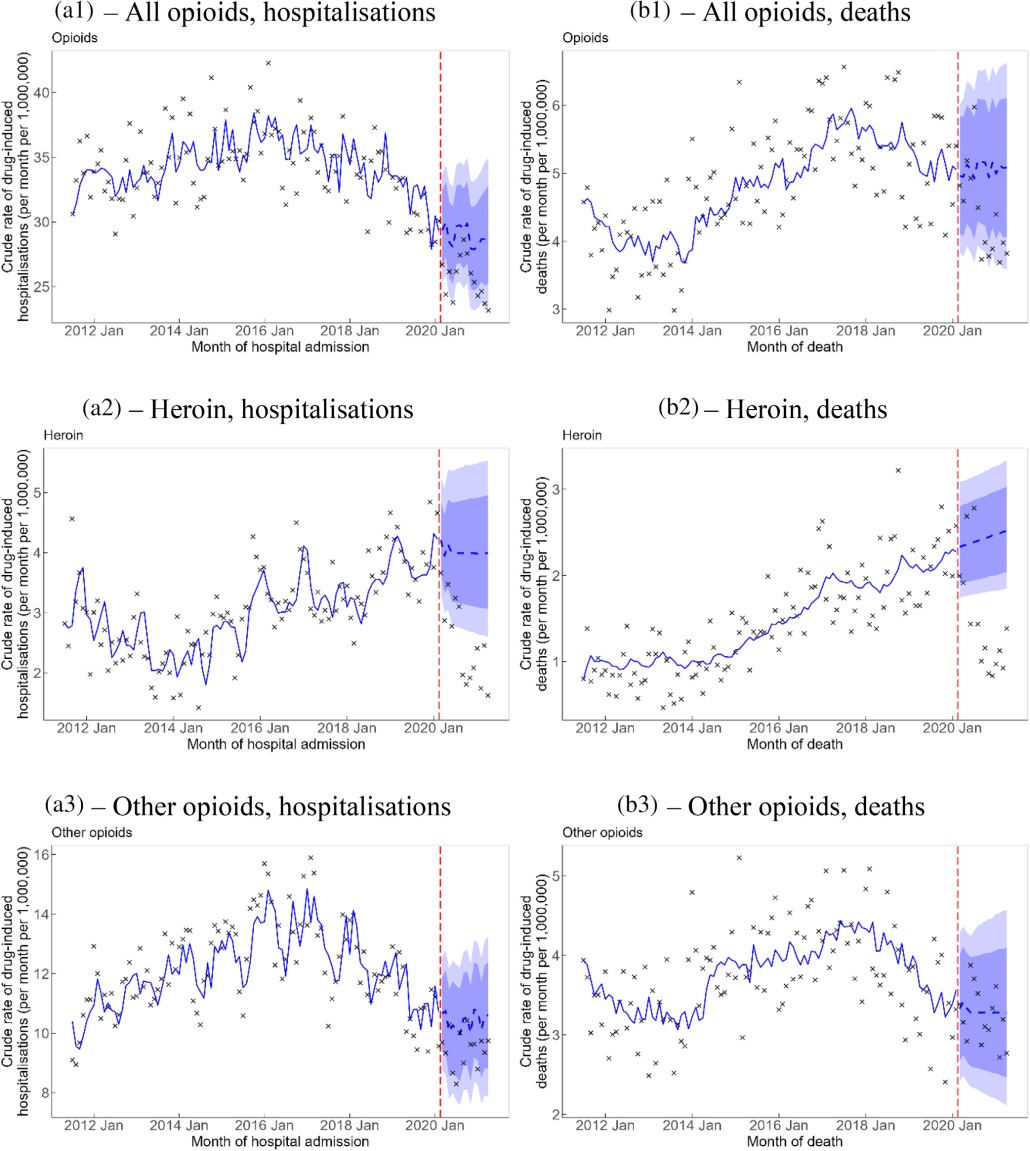
Crude rate of drug‐induced hospitalisations (A1–A3) and deaths (B1–B3) involving opioids. Vertical red dashed line represents the onset of the COVID‐19 pandemic. Crosses represent observed rates. Blue solid line represents the estimated trend and blue dashed line represents the counterfactual forecast of the trend after the onset of the COVID‐19 pandemic. Light blue and dark blue bands represent the 80% and 95% prediction intervals, respectively, of the counterfactual forecast of the rates after the onset of the COVID‐19 pandemic. ‘Other opioids’ exclude opium and heroin.

The observed rates of opioid‐related and heroin‐related hospitalisations during the COVID‐19 pandemic period were lower than forecasted by 3.3 (95% PI −6.3, −0.5) and 1.5 (95% PI −2.4, −0.7) hospitalisations per 1,000,000 persons per month, respectively (Table 1). Figure 3 shows that the decrease in rates of opioid‐related and heroin‐related hospitalisations were quite uniform over the COVID‐19 pandemic period during the study. This was more apparent for heroin, where all observed data were under the 80% PI. The observed rate of drug‐induced deaths involving heroin was lower than forecasted by 1.0 (95% PI −1.3, −0.6) deaths per 1,000,000 persons per month. The observed rate of drug‐induced deaths involving heroin was mostly within the 80% PI before August 2020, then dropped to a lower level when all the observed data points were under the 95% PI until the end of the study.

**FIGURE 4:**
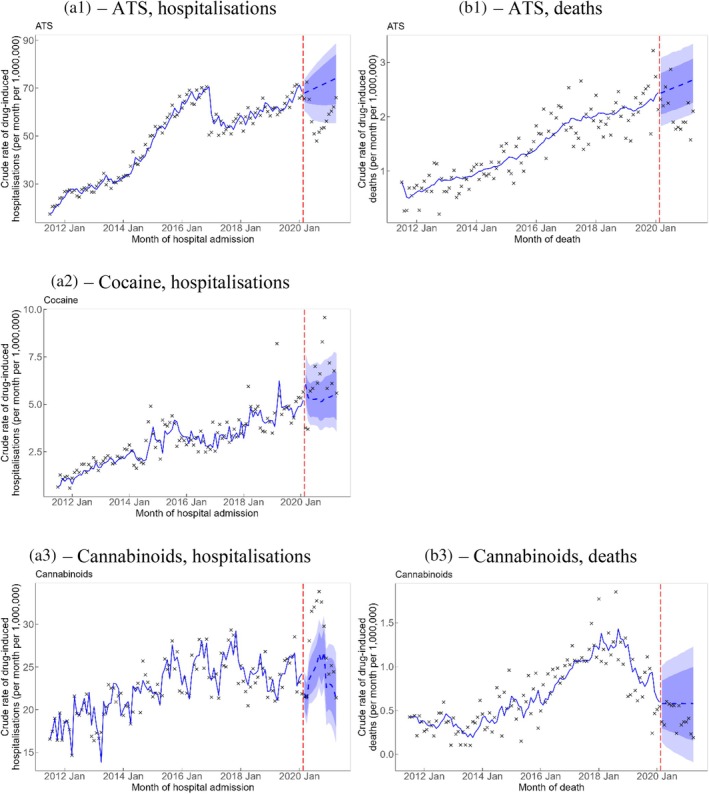
Crude rate of drug‐induced hospitalisations (A1–A3) and deaths (B1 and B3) involving amphetamine‐type stimulants (ATS), cocaine and cannabinoids. Vertical red dashed line represents the onset of the COVID‐19 pandemic. Crosses represent observed rates. Blue solid line represents the estimated trend and blue dashed line represents counterfactual forecast of the trend after the onset of the COVID‐19 pandemic. Light blue and dark blue bands represent the 80% and 95% prediction intervals, respectively, of counterfactual forecast of the rates after the onset of the COVID‐19 pandemic. (B2) is not shown because drug‐induced deaths involving cocaine were not analysed because the numbers were too small, that is, < 5 events in at least 1 month of data.

The observed rates of drug‐induced hospitalisation and death involving ATS were lower than predicted by 12.4 (−21.4, −0.8) hospitalisations and 0.5 (−0.7, −0.2) deaths per 1,000,000 persons per month, respectively. The counterfactual trend in Figure 4 evidenced a decrease in the ATS‐induced hospitalisation rates that troughed around the middle of 2020 and subsequently increased over time, such that the observed rates dropped below the 95% PI between June to around the end of 2020 and it was still under the 80% PI until almost the end of the study in April 2021. The observed rates of drug‐induced death involving ATS were mostly under the 95% PI from around August 2020 onwards.

The observed rate of cannabinoid‐related hospitalisations was higher than forecasted by 3.8 (95% PI 0.8, 6.8) hospitalisations per 1,000,000 persons per month (Table 1). This increase was most apparent in the first 6 months of the COVID‐19 pandemic from around May to November 2020 when the observed rates were above the 95% PI (Figure 4). No statistically significant changes were observed for any other drug classes for both hospitalisations and deaths.

### 3.4 Sensitivity Analyses

Results of sensitivity analysis for the time series from July 2016 with and without adjusting for all‐cause hospitalisation rates are presented in Appendix D in Supporting Information. In contrast to the longer time series, the results from the shorter time series without adjustment showed that the observed rates were higher than the forecasted rates among females and lower than the forecasted rates among people aged 35–54 and 55+ years, and the decrease in observed rates for ATS‐induced hospitalisations was no longer statistically significant. Significant changes in drug‐induced hospitalisations with adjustment for all‐cause hospitalisation rates were similar to those without adjustment from the shorter time series, except for among people aged 15–34 years, with an observed rate that was higher than forecasted by 21.4 (95% PI 1.4, 41.9) hospitalisations per 1,000,000 per month during the pandemic and among people aged 35–54 years of age, with no statistically significant change compared to the forecast.

## 4 Discussion

This study examined changes in drug‐induced harms in Australia during the first year of the COVID‐19 pandemic. Overall, observed rates of drug‐induced hospitalisation and death were not significantly different from the forecasted rates. While this was largely consistent across demographic groups, there were differences by drug involved, with evidence of a rise in the rate of cannabis‐induced hospitalisations and a decline in the rates of heroin and ATS‐induced hospitalisations and deaths.

Our observation that overall rates of drug‐induced harms did not change during the pandemic and rates of ATS and heroin‐induced harms declined is counter to the observation of increased drug‐related deaths in Canada and England [10, 11, 35]. This occurred in the context of worsening mental health among the Australian population [15] and difficulties accessing related services [20, 21] but aligns with findings that heroin‐related ambulance attendances decreased in Victoria [22] and overdoses decreased at medically supervised injecting sites [21]. Australian heroin and methamphetamine markets experienced disruptions in 2020 [17] and surveys with people who inject drugs indicated that some people reduced their use of these drugs in response to lower availability and higher prices [19]. Purity of these drugs was also perceived to have decreased [17]. Indeed, a decline in heroin and methamphetamine load in wastewater was reported [36], which is indicative of decreased use and/or decreased purity. Drug markets are a known determinant of use and harms [37] and a heroin shortage in Australia that began in early 2001 also led to a decline in overdose deaths [38]. The increased vulnerability of Australian illicit drug markets to disruption compared to England and North America as well as the existing overdose crisis in North America [12] that may have been exacerbated by pandemic‐related stressors and impacts to healthcare provision may explain our contrasting observations. Moreover, access to opioid agonist treatment in Australia during the pandemic was improved, with an increase in the number of clients receiving take‐home doses and depot buprenorphine [39]. With pandemic‐associated restrictions lifted as of late 2022, ongoing monitoring of drug‐induced harms is essential to discern whether reductions in harms were transient.

In contrast, we observed an increase in cannabinoid‐induced hospitalisations. Australia's cannabis market was relatively unaffected by restrictions in 2020 and 2021 [40, 41], potentially because it is domestically produced and thus more resistant to supply chain disruptions. Indeed, consistent with evidence from Europe [42], sentinel surveys of people who use drugs in Australia showed that many people reported stable or increased cannabis use in early 2020 compared to pre‐pandemic. This is also reflected in wastewater data, which indicated an increased load of cannabis during the pandemic [43]. These observations are similar to those for alcohol. During the pandemic, alcohol use increased among some populations in Australia [44] and an excess of alcohol‐induced hospitalisations and deaths was observed [45]. Already the two most commonly used drugs in Australia (excluding tobacco) prior to the pandemic [46], alcohol and cannabis were likely more readily accessible and easier to use in response to the various stressors associated with the pandemic and associated restrictions.

Consistent with our overall findings, all demographic groups (i.e., by age and sex) appeared to experience no rise in rates of hospitalisations. We also found either no significant change or a significant decrease in rates of drug‐induced deaths for all demographic groups. This points to a decrease in fatal overdose during the pandemic among most demographic cohorts, likely relating to reduced access and opportunity to use drugs as outlined above [40, 42, 47].

Current evidence indicates there was an overall downwards trend in healthcare service utilisation globally during COVID‐19 [48], meaning some people who may have benefited from hospital care were potentially unable or unwilling to access it. Our sensitivity analysis adjusted for all‐cause hospitalisation rates [48, 49] and showed a significant increase in drug‐induced hospitalisation among people aged 15–34 years. While there was also a significant increase in drug‐induced hospitalisations among females and a significant decrease among people aged 55+ years after adjusting for all‐cause hospitalisations, they were also statistically significant in the same direction in the corresponding shorter time series without the adjustment.

Continued monitoring and a deeper exploration of factors driving drug‐induced harms are warranted. Although evidence from three Australian states suggests there was no overall increase in suicide deaths during the pandemic [50], demographic profiles of intentional and unintentional drug‐induced deaths are distinct [51], so future research could examine differential changes in intentional and unintentional deaths during the pandemic. Many people experienced access difficulties and fewer opportunities to use drugs during lockdown periods [52], and harms relating to these drugs may increase as restrictions ease. Both heroin and methamphetamine markets showed strong recovery by late 2021 [17, 36]; it is as yet unclear whether this may have precipitated a rise in harms. It is also important to consider broader changes to policy and practice coinciding with the pandemic, which may have contributed to the reduction in drug‐induced hospitalisations and deaths we observed. For example, Australia introduced a pilot to improve naloxone access in three states (New South Wales, Western Australia and South Australia) in December 2019 that continued throughout the study period [53]. Ongoing monitoring is crucial to determine whether short‐term trends revert with the removal of restrictions in Australia in 2021–22, or whether the effects are longer‐lasting.

### 4.1 Limitations

This study had several limitations. First, data should be interpreted with caution given the widespread impacts of pandemic‐related restrictions on service provision (e.g., in hospitals). However, we performed a sensitivity analysis on drug‐induced hospitalisation rates adjusting for all‐cause hospitalisation to assess the potential impact of service provision [48, 49]. Trends in absolute numbers may differ from reported trends in rates due to net negative migration during the pandemic. Australia experienced a net loss of 89,000 people during the 2020–21 financial year, affecting every state and territory [54]. Indeed, the reported rates in the present study likely reflect higher relative increases and lower relative decreases compared with results using numbers. There was state and territory variation in public health measures in both duration and stringency, but separate analyses for each state and territory were precluded by sparse numbers, particularly for the smaller states and territories. We could not conduct analyses of polysubstance use due to small numbers reporting specific profiles of use. Identification of drug involvement is typically dependent on toxicological testing for the substance; given practices vary by jurisdiction and over time, there may be some under‐ascertainment of cases.

## 5 Conclusion

We found no overall evidence of an increase in drug‐induced harms during the COVID‐19 pandemic. There were some differences by the drug involved, with evidence of a decrease in drug‐induced hospitalisations and deaths involving heroin and ATS, and an increase in cannabinoid‐induced hospitalisations. These may be explained by disruption to some illicit drug markets in Australia. Ongoing monitoring remains important to understand whether the changes we observed with respect to some drug‐induced harms endure.

## Author Contributions

A.P., N.M. and A.C. conceived the study. All authors had input on the study design. N.M. and A.C. prepared the data for analysis. N.M. conducted analyses with support from A.C. N.M., J.A., O.P., A.C. and A.P. drafted the manuscript. All authors had input on revising the manuscript critically for important intellectual content. All authors approved the final version to be published. Each author certifies that their contribution to this work meets the standards of the International Committee of Medical Journal Editors.

## Conflicts of Interest

A.P. has received untied educational grants from Seqirus and Mundipharma for post‐marketing surveillance of pharmaceutical opioids. R.S. has received untied educational grants from Seqirus for post‐marketing surveillance of pharmaceutical opioids. L.D. discloses untied educational grants from Seqirus, Indivior, and Mundipharma for the study of opioid medications. These grants have ceased, and the funders had no role in study design, conduct or reporting. No pharmaceutical grants were received for the current study. All other authors have no conflicts of interest to declare.

## Supporting information


Data S1.

## Data Availability

Authorisation for the use of National Hospital Morbidity Database data was obtained from the Australian Institute of Health and Welfare. Authorisation for the use of the Cause of Death Unit Record Files was obtained from the Australian Bureau of Statistics. The authors are unable to share these data due to ethics restrictions.
