# Supplementary material for: Changes in Drug‐Induced Hospitalisations and Deaths During the First Year of the COVID‐19 Pandemic in Australia
Source: Drug Alcohol Rev. 2025 Jun 8;44(5):1419–29. doi: 10.1111/dar.14088 (PMC12228029; doi:10.1111/dar.14088)

**SUPPLEMENTARY MATERIAL FOR “CHANGES IN DRUG-INDUCED HOSPITALISATIONS AND DEATHS FOLLOWING THE ONSET OF THE COVID-19 PANDEMIC IN AUSTRALIA”**

[Appendix A. STROBE Checklist 2](#_Toc193122422)

[Appendix B. ICD Codes 5](#_Toc193122423)

[Appendix C. Statistical tests and plots for assumptions of the ARIMA models 9](#_Toc193122424)

[Appendix D. Results using the shorter time series from 1^st^ July 2016 to 30^th^ April 2021, with and without adjusting for all-cause hospitalisation rates 23](#_Toc193122425)

# Appendix A. STROBE Checklist

STROBE Statement—Checklist of items that should be included in reports of ***cross-sectional studies***

|  | Item No | Recommendation | Complete |
| --- | --- | --- | --- |
| **Title and abstract** | 1 | (*a*) Indicate the study’s design with a commonly used term in the title or the abstract | ✓ |
|  |  | (*b*) Provide in the abstract an informative and balanced summary of what was done and what was found | ✓ |
| Introduction | | |  |
| Background/rationale | 2 | Explain the scientific background and rationale for the investigation being reported | ✓ |
| Objectives | 3 | State specific objectives, including any prespecified hypotheses | ✓ |
| Methods | | |  |
| Study design | 4 | Present key elements of study design early in the paper | ✓ |
| Setting | 5 | Describe the setting, locations, and relevant dates, including periods of recruitment, exposure, follow-up, and data collection | ✓ |
| Participants | 6 | (*a*) Give the eligibility criteria, and the sources and methods of selection of participants | ✓ |
| Variables | 7 | Clearly define all outcomes, exposures, predictors, potential confounders, and effect modifiers. Give diagnostic criteria, if applicable | ✓ |
| Data sources/ measurement | 8* | For each variable of interest, give sources of data and details of methods of assessment (measurement). Describe comparability of assessment methods if there is more than one group | ✓ |
| Bias | 9 | Describe any efforts to address potential sources of bias | ✓ |
| Study size | 10 | Explain how the study size was arrived at | N.A. (given secondary data sources) |
| Quantitative variables | 11 | Explain how quantitative variables were handled in the analyses. If applicable, describe which groupings were chosen and why | ✓ |
| Statistical methods | 12 | (*a*) Describe all statistical methods, including those used to control for confounding | ✓ |
|  |  | (*b*) Describe any methods used to examine subgroups and interactions | N.A. |
|  |  | (*c*) Explain how missing data were addressed | N.A. |
|  |  | (*d*) If applicable, describe analytical methods taking account of sampling strategy | ✓ |
|  |  | (*e*) Describe any sensitivity analyses | ✓ |
| Results | | |  |
| Participants | 13* | (a) Report numbers of individuals at each stage of study—eg numbers potentially eligible, examined for eligibility, confirmed eligible, included in the study, completing follow-up, and analysed | ✓ |
|  |  | (b) Give reasons for non-participation at each stage | N.A. |
|  |  | (c) Consider use of a flow diagram | N.A. |
| Descriptive data | 14* | (a) Give characteristics of study participants (eg demographic, clinical, social) and information on exposures and potential confounders | ✓ |
|  |  | (b) Indicate number of participants with missing data for each variable of interest | N.A. |
| Outcome data | 15* | Report numbers of outcome events or summary measures | ✓ |
| Main results | 16 | (*a*) Give unadjusted estimates and, if applicable, confounder-adjusted estimates and their precision (eg, 95% confidence interval). Make clear which confounders were adjusted for and why they were included | ✓ |
|  |  | (*b*) Report category boundaries when continuous variables were categorized | N.A. |
|  |  | (*c*) If relevant, consider translating estimates of relative risk into absolute risk for a meaningful time period | N.A. |
| Other analyses | 17 | Report other analyses done—eg analyses of subgroups and interactions, and sensitivity analyses | ✓ |
| Discussion | | |  |
| Key results | 18 | Summarise key results with reference to study objectives | ✓ |
| Limitations | 19 | Discuss limitations of the study, taking into account sources of potential bias or imprecision. Discuss both direction and magnitude of any potential bias | ✓ |
| Interpretation | 20 | Give a cautious overall interpretation of results considering objectives, limitations, multiplicity of analyses, results from similar studies, and other relevant evidence | ✓ |
| Generalisability | 21 | Discuss the generalisability (external validity) of the study results | ✓ |
| Other information | | |  |
| Funding | 22 | Give the source of funding and the role of the funders for the present study and, if applicable, for the original study on which the present article is based | ✓ |

# Appendix B. ICD Codes

**NHMD:** Hospitalisations for which the care type was reported as 'Newborn without qualified days’, and records for ‘Posthumous organ procurement’ and 'Hospital boarders’ were not provided.

**Table B1. ICD-10-AM codes in principal diagnosis to identify drug-induced hospitalisations**

| **ICD-10-AM code** | **Description** |
| --- | --- |
| F11 | Mental and behavioural disorders due to use of opioids |
| F12 | Mental and behavioural disorders due to use of cannabinoids |
| F13 | Mental and behavioural disorders due to use of sedatives or hypnotics |
| F14 | Mental and behavioural disorders due to use of cocaine |
| F15 | Mental and behavioural disorders due to use of other stimulants, including caffeine |
| F16 | Mental and behavioural disorders due to use of hallucinogens |
| F18 | Mental and behavioural disorders due to use of volatile solvents |
| F19 | Mental and behavioural disorders due to multiple drug use and use of other psychoactive substances |
| F55.0 | Harmful use of nondependence-producing substance, antidepressants |
| F55.2 | Harmful use of nondependence-producing substance, analgesics |
| N14.0 | Drug- and heavy-metal-induced tubulo-interstitial and tubular conditions: Analgesic nephropathy |
| T39.0–T39.9 | Poisoning by nonopioid analgesics, antipyretics and antirheumatics |
| T40.0-T40.9 | Poisoning by narcotics and psychodysleptics [hallucinogens] |
| T41.2 | Poisoning by anaesthetics and therapeutic gases: Other and unspecified general anaesthetics |
| T42.0–T42.8 | Poisoning by antiepileptic, sedative-hypnotic and antiparkinsonism drugs |
| T43.0-T43.6 | Poisoning by (specified) psychotropic drugs, |
| T52.0–T52.9 | Toxic effect of organic solvents |
| T53.0–T53.9 | Toxic effect of halogen derivatives of aliphatic and aromatic hydrocarbons |
| T59.0, T59.8 | Toxic effect of other gases, fumes and vapours: Nitrogen oxides and Other specified gases, fumes and vapours |

**Table B2. ICD-10 codes for underlying causes of death to identify drug-induced mortality**

| **ICD-10 codes** | **Description** |
| --- | --- |
| D52.1 | Drug-induced folate deficiency anaemia |
| D59.0 | Drug-induced haemolytic anaemia |
| D59.2 | Drug-induced nonautoimmune haemolytic anaemia |
| D61.1 | Drug-induced aplastic anaemia |
| D64.2 | Secondary sideroblastic anaemia due to drugs and toxins |
| E06.4 | Drug-induced thyroiditis |
| E16.0 | Drug-induced hypoglycaemia without coma |
| E23.1 | Drug-induced hypopituitarism |
| E24.2 | Drug-induced Cushing’s syndrome |
| E27.3 | Drug-induced adrenocortical insufficiency |
| E66.1 | Drug-induced obesity |
| F11.0-F11.5 | Use of opioids causing intoxication harmful use (abuse) dependence withdrawal or psychosis |
| F11.7-F11.9 | Use of opioid causing late onset psychosis other mental and behavioural disorders and unspecified behavioural disorders |
| F12.0-F12.5 | Use of cannabis causing intoxication harmful use (abuse) dependence withdrawal or psychosis |
| F12.7-F12.9 | Use of cannabis causing late onset psychosis other mental and behavioural disorders and unspecified behavioural disorders. |
| F13.0-F13.5 | Use of sedative or hypnotics causing intoxication harmful use (abuse) dependence withdrawal or psychosis |
| F13.7-F13.9 | Use of sedative or hypnotics causing late onset psychosis other mental and behavioural disorders and unspecified behavioural disorders |
| F14.0-F14.5 | Use of cocaine causing intoxication harmful use (abuse) dependence withdrawal or psychosis |
| F14.7-F14.9 | Use of cocaine causing late onset psychosis other mental and behavioural disorders and unspecified behavioural disorders |
| F15.0-F15.5 | Use of caffeine causing intoxication harmful use (abuse) dependence withdrawal or psychosis |
| F15.7-F15.9 | Use of caffeine causing late onset psychosis other mental and behavioural disorders and unspecified behavioural disorders |
| F16.0-F16.5 | Use of hallucinogens causing intoxication harmful use (abuse) dependence withdrawal or psychosis |
| F16.7-F16.9 | Use of hallucinogens causing late onset psychosis other mental and behavioural disorders and unspecified behavioural disorders |
| F18.0-F18.5 | Use of volatile solvents causing intoxication harmful use (abuse) dependence withdrawal or psychosis |
| F18.7-F18.9 | Use of volatile solvents causing late onset psychosis other mental and behavioural disorders and unspecified behavioural disorders |
| F19.0-F19.5 | Use of multiple drugs and other psychoactive substances causing intoxication harmful use (abuse) dependence withdrawal or psychosis |
| F19.7-F19.9 | Use of multiple drugs and other psychoactive substances causing late onset psychosis other mental and behavioural disorders and unspecified behavioural disorders |
| G21.1 | Other drug-induced secondary Parkinsonism |
| G24.0 | Drug-induced dystonia |
| G25.1 | Drug-induced tremor |
| G25.4 | Drug-induced chorea |
| G25.6 | Drug-induced tics and other tics of organic origin |
| G44.4 | Drug-induced headache not elsewhere classified |
| G62.0 | Drug-induced polyneuropathy |
| G72.0 | Drug-induced myopathy |
| I95.2 | Hypotension due to drugs |
| J70.2 | Acute drug-induced interstitial lung disorders |
| J70.3 | Chronic drug-induced interstitial lung disorders |
| J70.4 | Drug-induced interstitial lung disorder unspecified |
| L10.5 | Drug-induced pemphigus |
| L27.0 | Generalized skin eruption due to drugs and medicaments |
| L27.1 | Localized skin eruption due to drugs and medicaments |
| M10.2 | Drug-induced gout |
| M32.0 | Drug-induced systemic lupus erythematosus |
| M80.4 | Drug-induced osteoporosis with pathological fracture |
| M81.4 | Drug-induced osteoporosis |
| M83.5 | Other drug-induced osteomalacia in adults |
| M87.1 | Osteonecrosis due to drugs |
| R78.1 | Finding of opiate drug in blood |
| R78.2 | Finding of cocaine in blood |
| R78.3 | Finding of hallucinogen in blood |
| R78.4 | Finding of other drugs of addictive potential in blood |
| R78.5 | Finding of psychotropic drug in blood |
| X40-X44 | Accidental poisoning by and exposure to drugs medicaments and biological substances |
| X60-X64 | Intentional self-poisoning (suicide) by and exposure to drugs medicaments and biological substances |
| X85 | Assault (homicide) by drugs medicaments and biological substances |
| Y10-Y14 | Poisoning by and exposure to drugs medicaments and biological substances undetermined intent |

Drug-induced causes exclude accidents, homicides, and other causes indirectly related to drug use. Also excluded are newborn deaths associated with mother’s drug use.

**Table B3. Drug groups and corresponding ICD-10-AM and ICD-10 codes used to identify the drug involved in drug-induced hospitalisations and deaths.**

|  |  | **Hospitalisations with the selected ICD-10-AM codes in the principal diagnosis** | **Drug-induced deaths with selected ICD-10 codes in any of the associated causes of death** |
| --- | --- | --- | --- |
| **Drug groups** | **Drugs included** | **ICD-10-AM** | **ICD-10** |
| Opioids |  | F11, T40.0-T40.4, T40.6 | T40.0-T40.4, T40.6 |
| Heroin | Heroin | T40.1 | T40.1 |
| Other opioids | Natural and semi-synthetic opioids (e.g., oxycodone, morphine), methadone, other synthetic opioids, other and unspecified opioids/narcotics (e.g., fentanyl, tramadol) | T40.2-T40.4, T40.6 | T40.2-T40.4, T40.6 |
| Amphetamine-type stimulants | Amphetamine, methamphetamine, MDMA/ecstasy, caffeine | F15, T43.6 | T43.6 |
| Cocaine | Cocaine | F14, T40.5 | T40.5 |
| Cannabinoids | Cannabis | F12, T40.7 | T40.7 |

# Appendix C. Statistical tests and plots for assumptions of the ARIMA models

**Table C1. Statistical tests for assumptions of the ARIMA models**

|  | **KPSS test** | | | |
| --- | --- | --- | --- | --- |
|  | **Drug-induced hospitalisations** | | **Drug-induced deaths** | |
| Overall | 0.191 | 0.100 | 0.130 | 0.100 |
| **Sex** |  |  |  |  |
| Males | 0.183 | 0.100 | 0.103 | 0.100 |
| Females | 0.334 | 0.100 | 0.117 | 0.100 |
| **Age** |  |  |  |  |
| 15-34 | 0.128 | 0.100 | 0.078 | 0.100 |
| 35-54 | 0.187 | 0.100 | 0.155 | 0.100 |
| 55+ | 0.256 | 0.100 | 0.283 | 0.100 |
| **Drug involved** |  |  |  |  |
| Opioids | 0.383 | 0.084 | 0.138 | 0.100 |
| Heroin | 0.161 | 0.100 | 0.153 | 0.100 |
| Other opioids | 0.395 | 0.079 | 0.215 | 0.100 |
| Amphetamine-type stimulants | 0.126 | 0.100 | 0.095 | 0.100 |
| Cocaine | 0.186 | 0.100 | n.p. | n.p. |
| Cannabinoids | 0.046 | 0.100 | 0.252 | 0.100 |

Note: ‘Other opioids’ exclude opium and heroin. n.p. = not published because the number of drug-induced deaths involving cocaine were too small, i.e. <5 events in at least one month of data.

ARIMA, autoregressive integrated moving average.

**Figure C1. Residual plots for drug-induced hospitalisations. ARIMA, autoregressive integrated moving average; ATS, amphetamine-type stimulants**
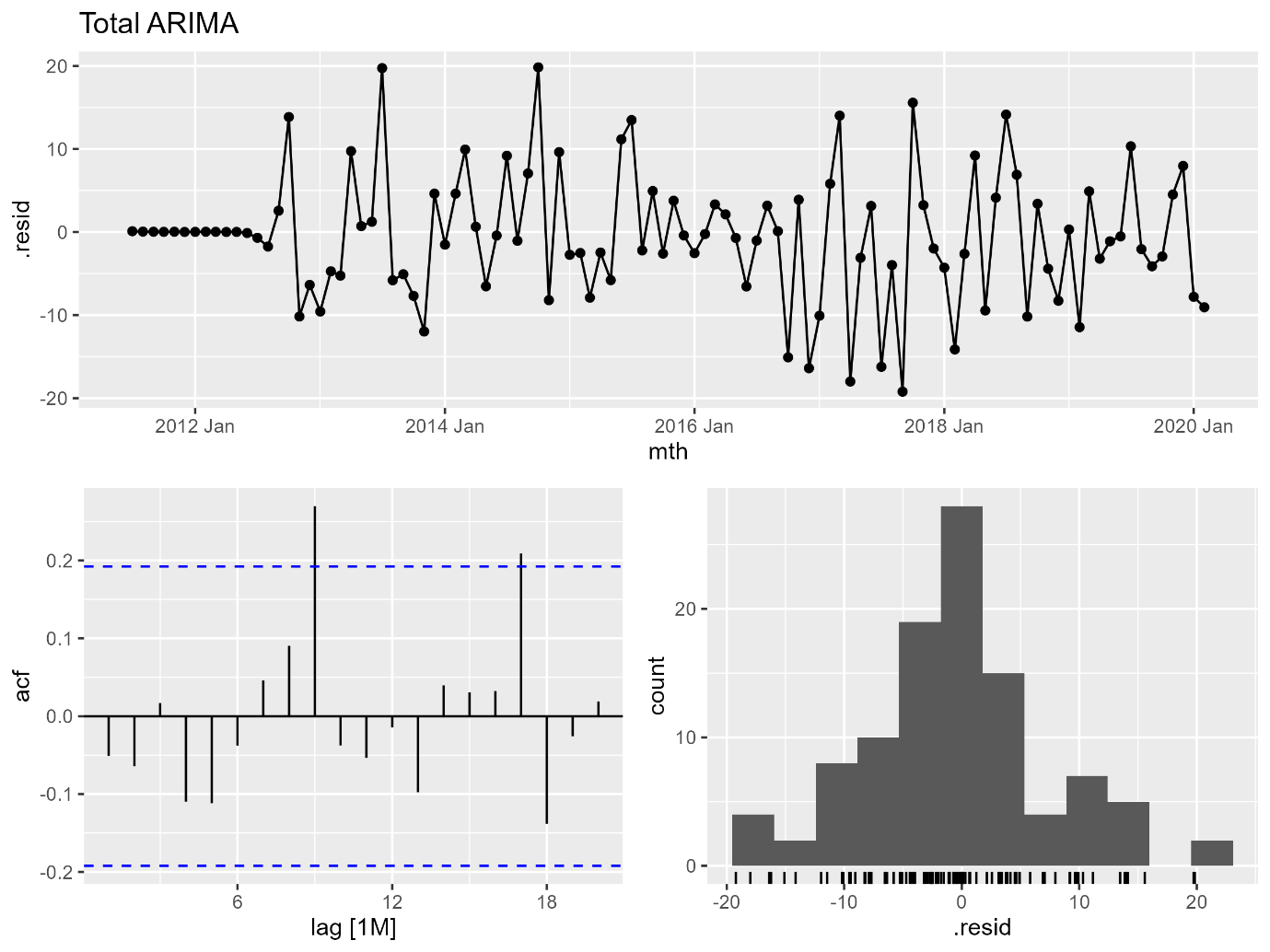

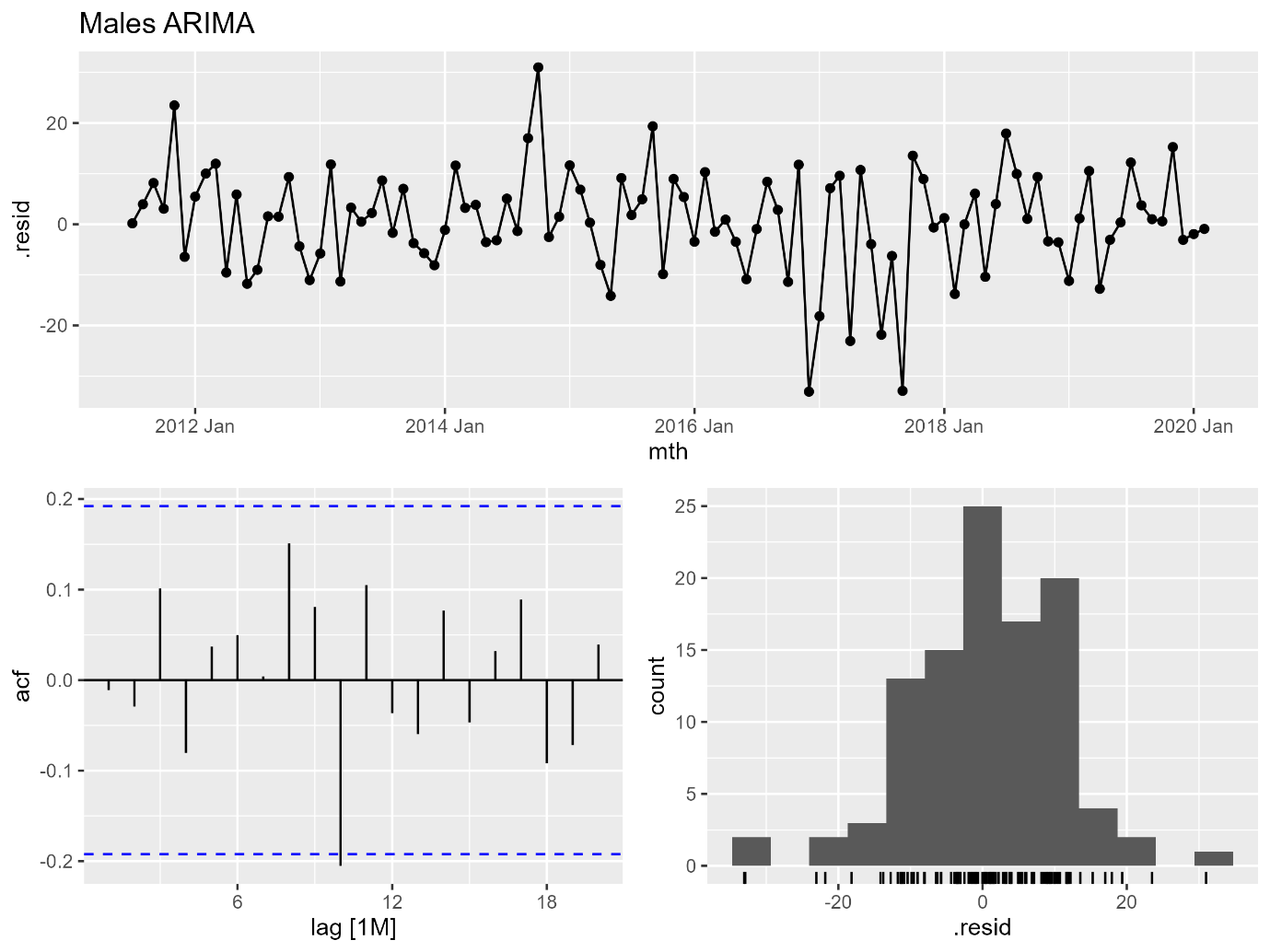


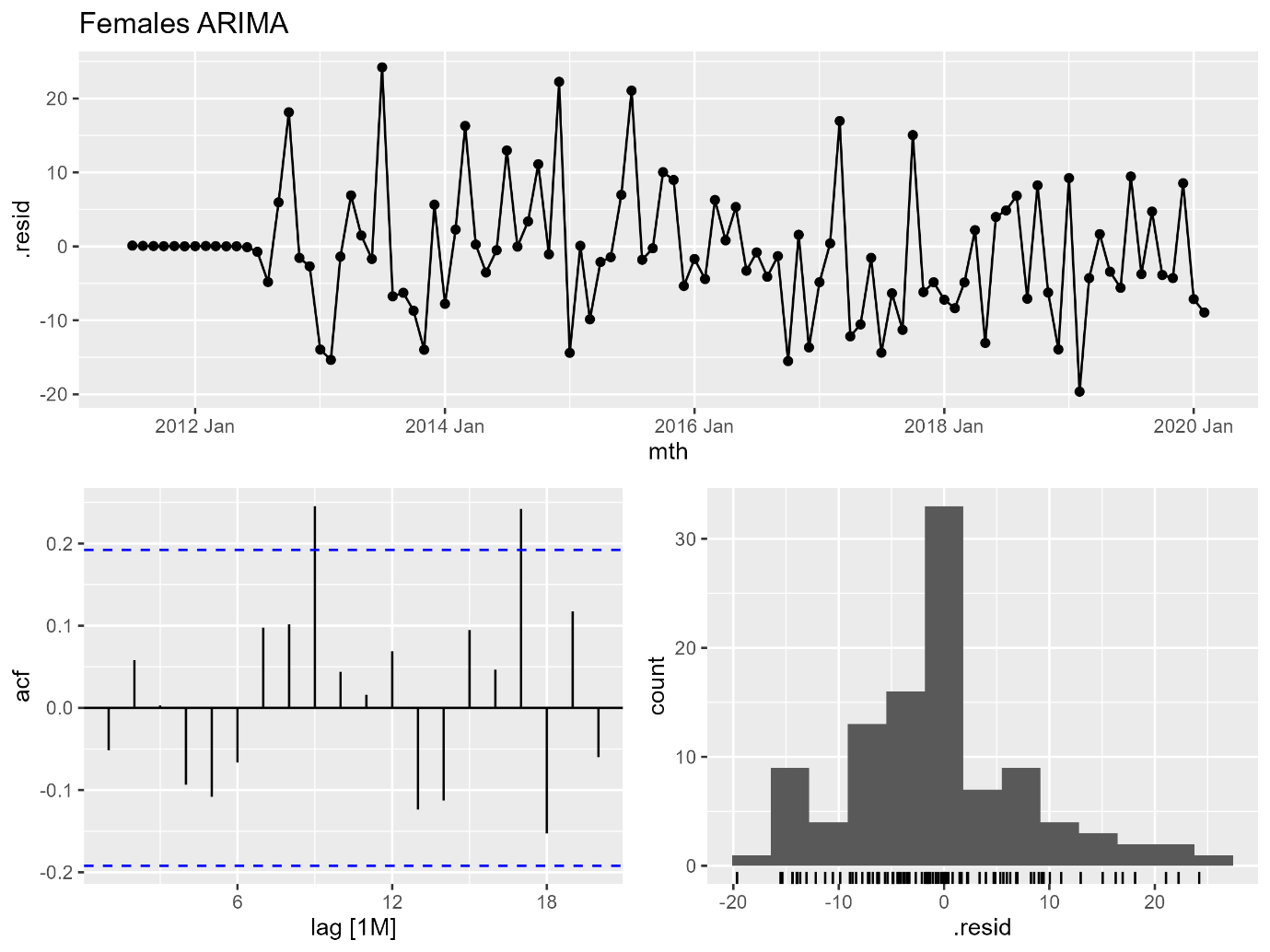


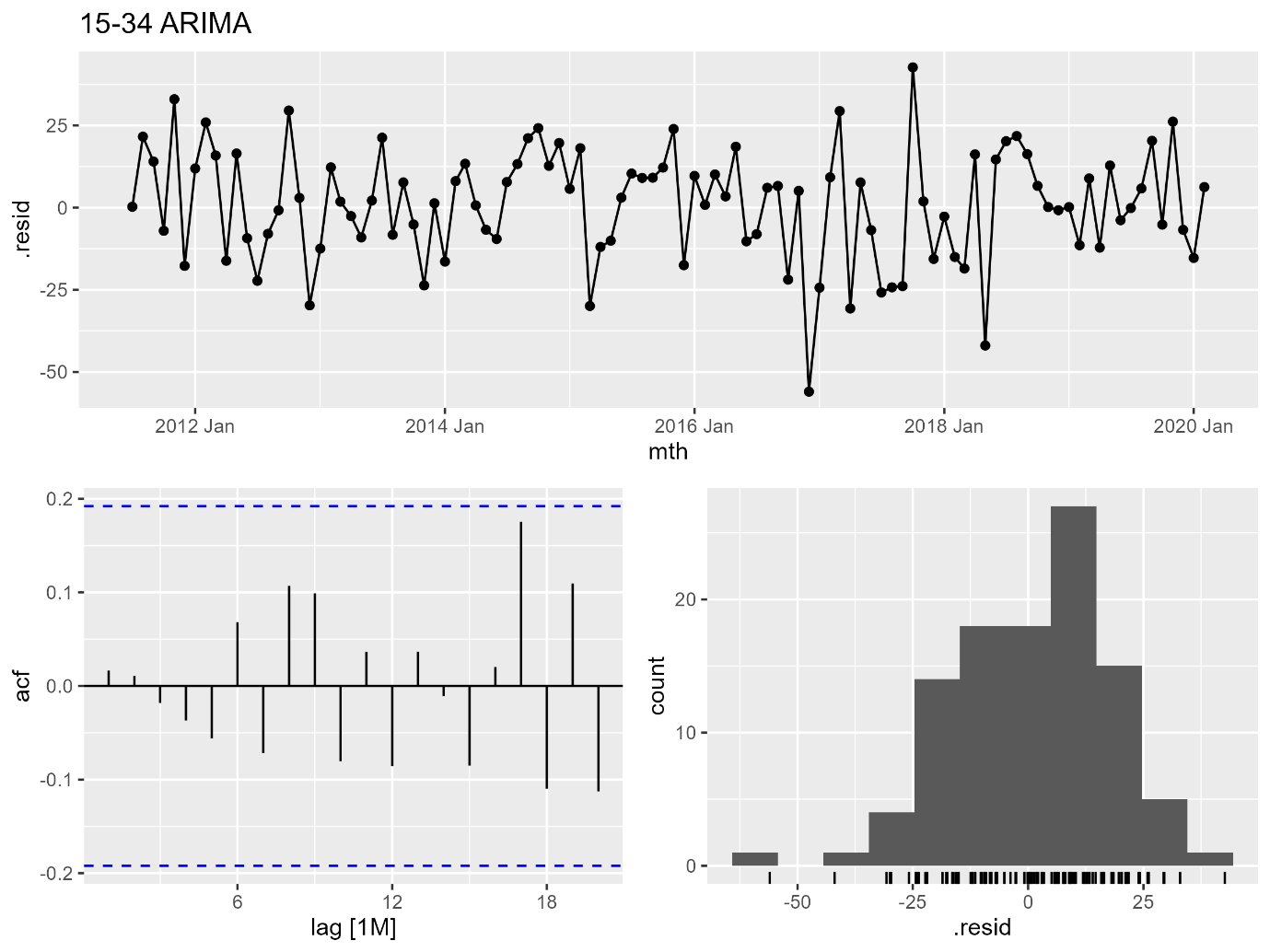


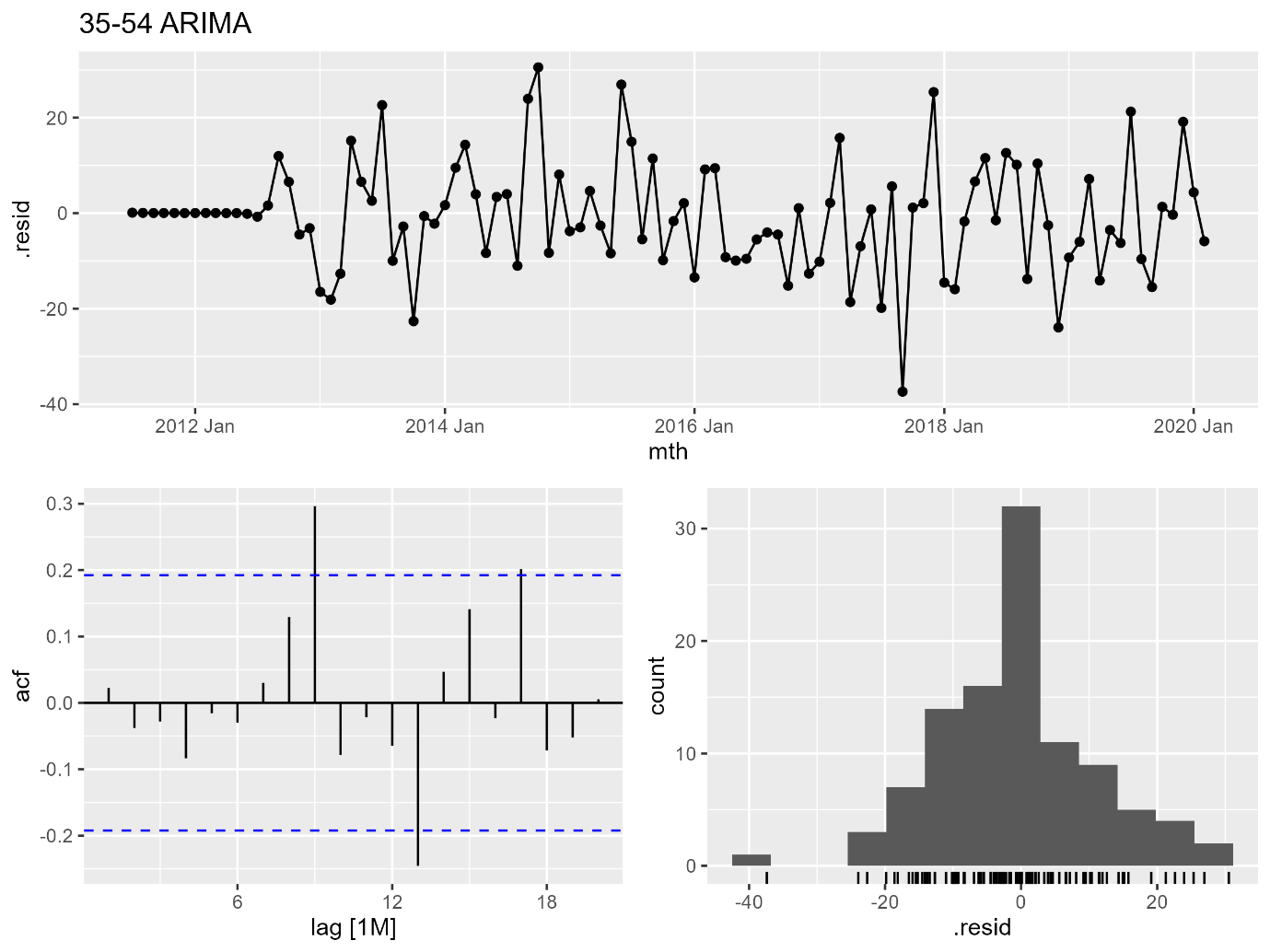


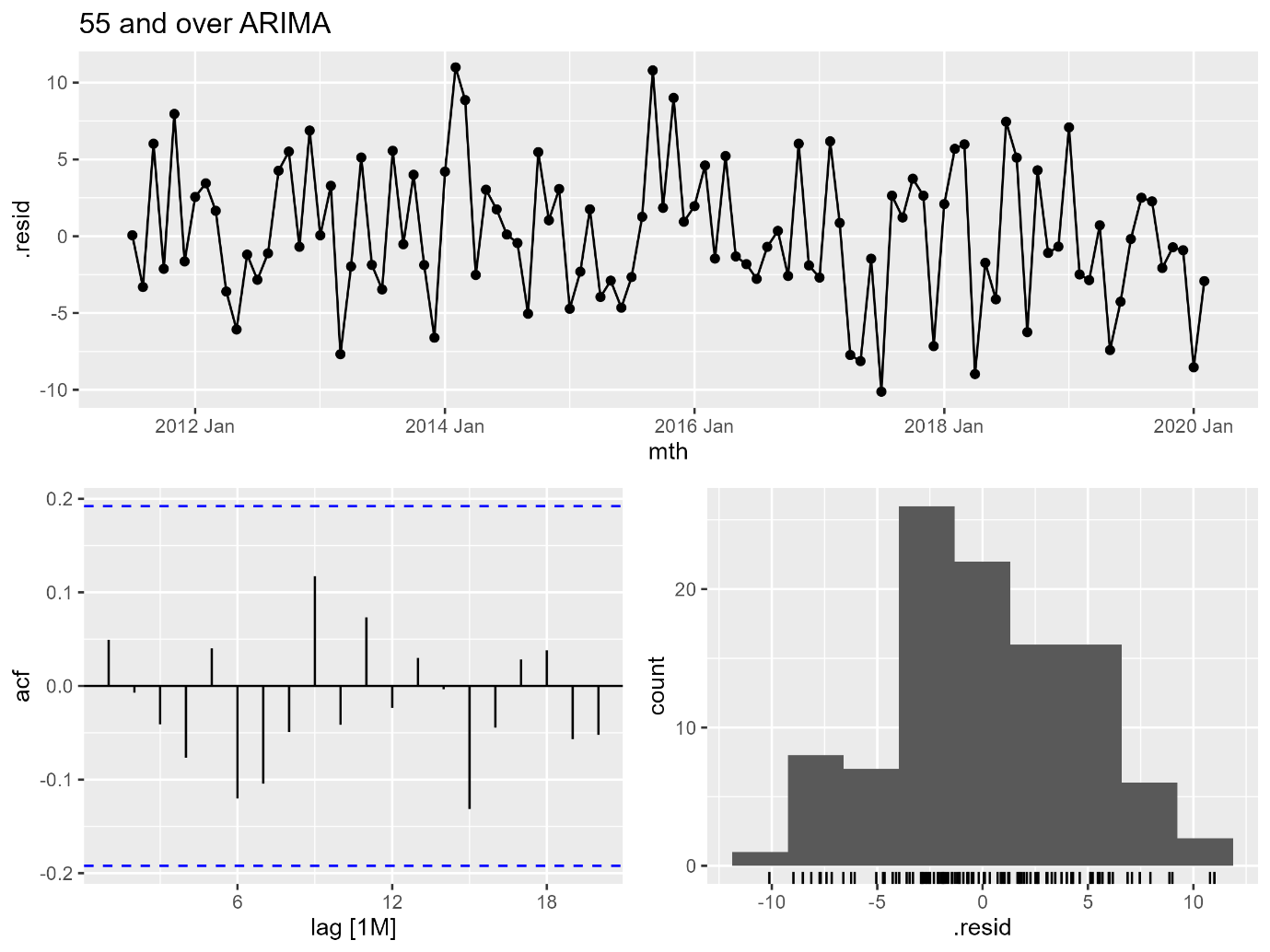


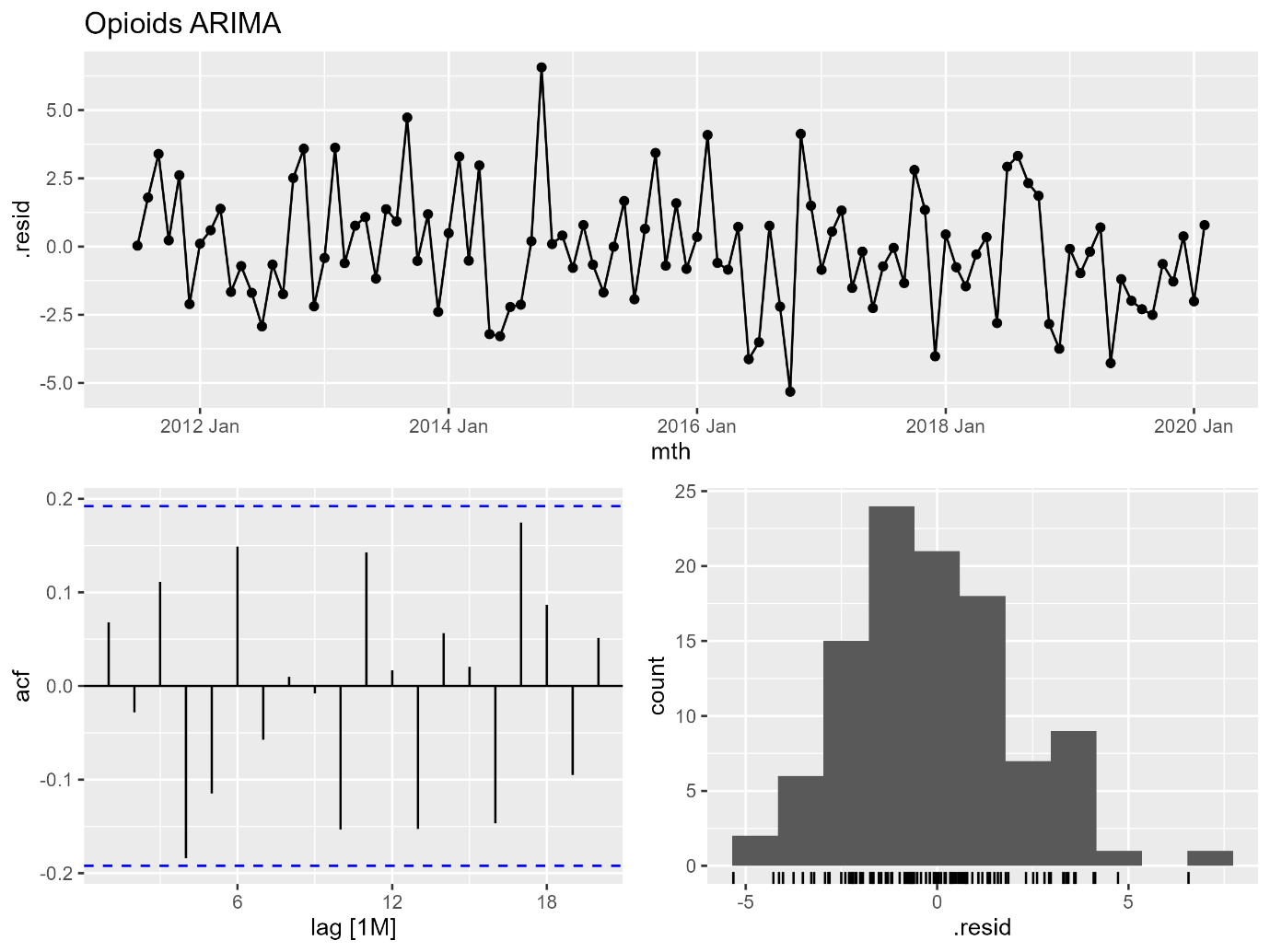


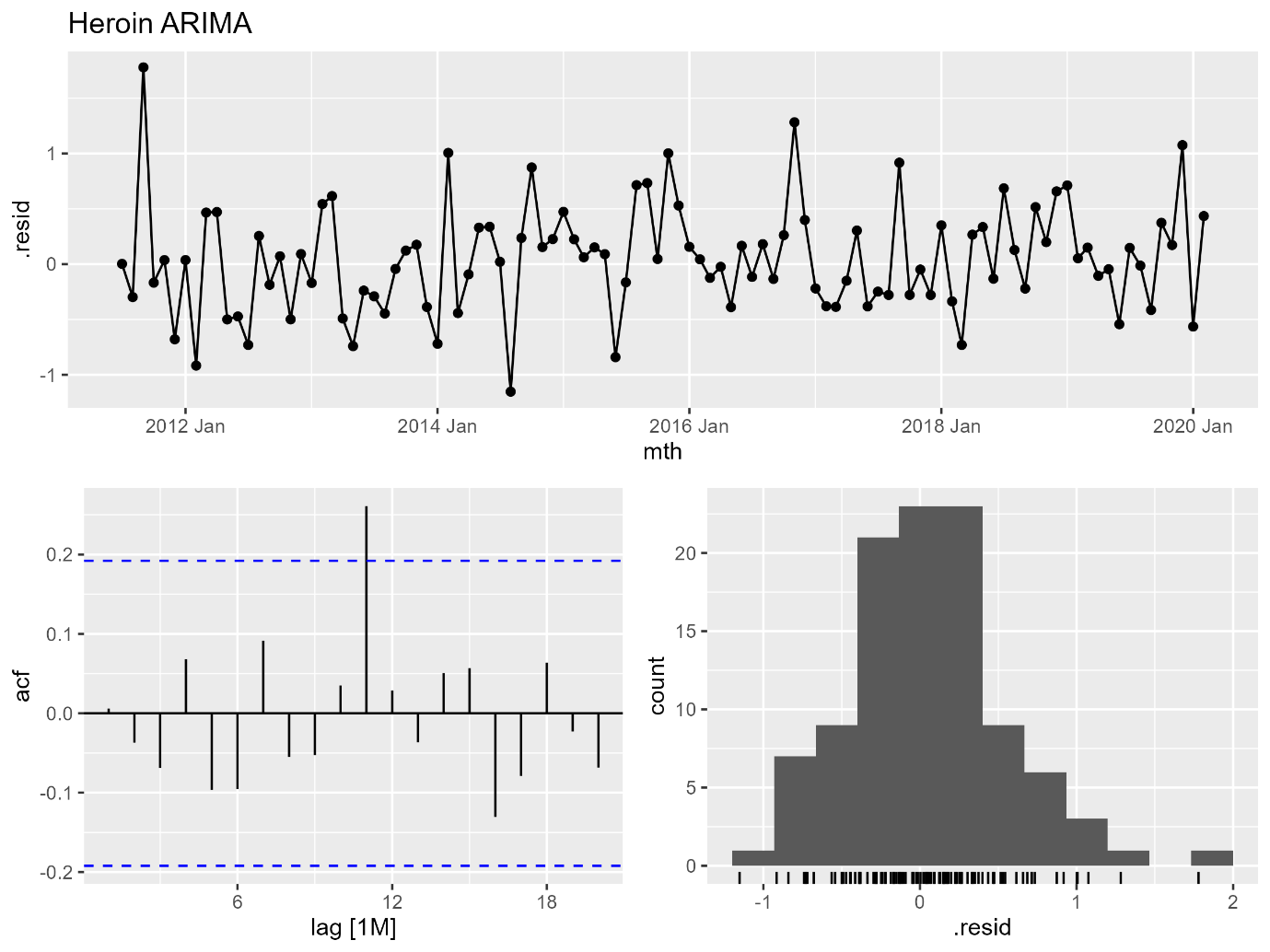


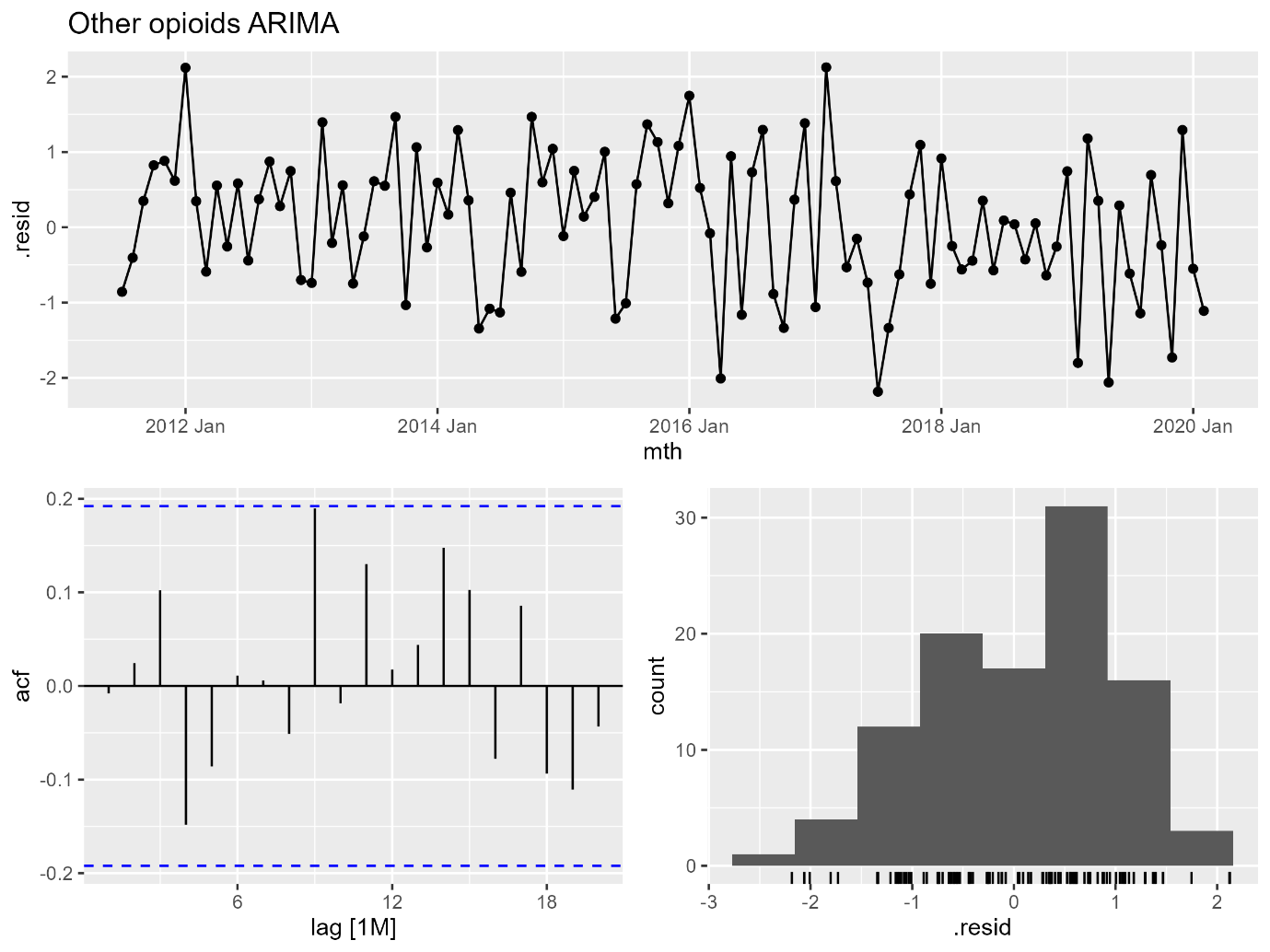


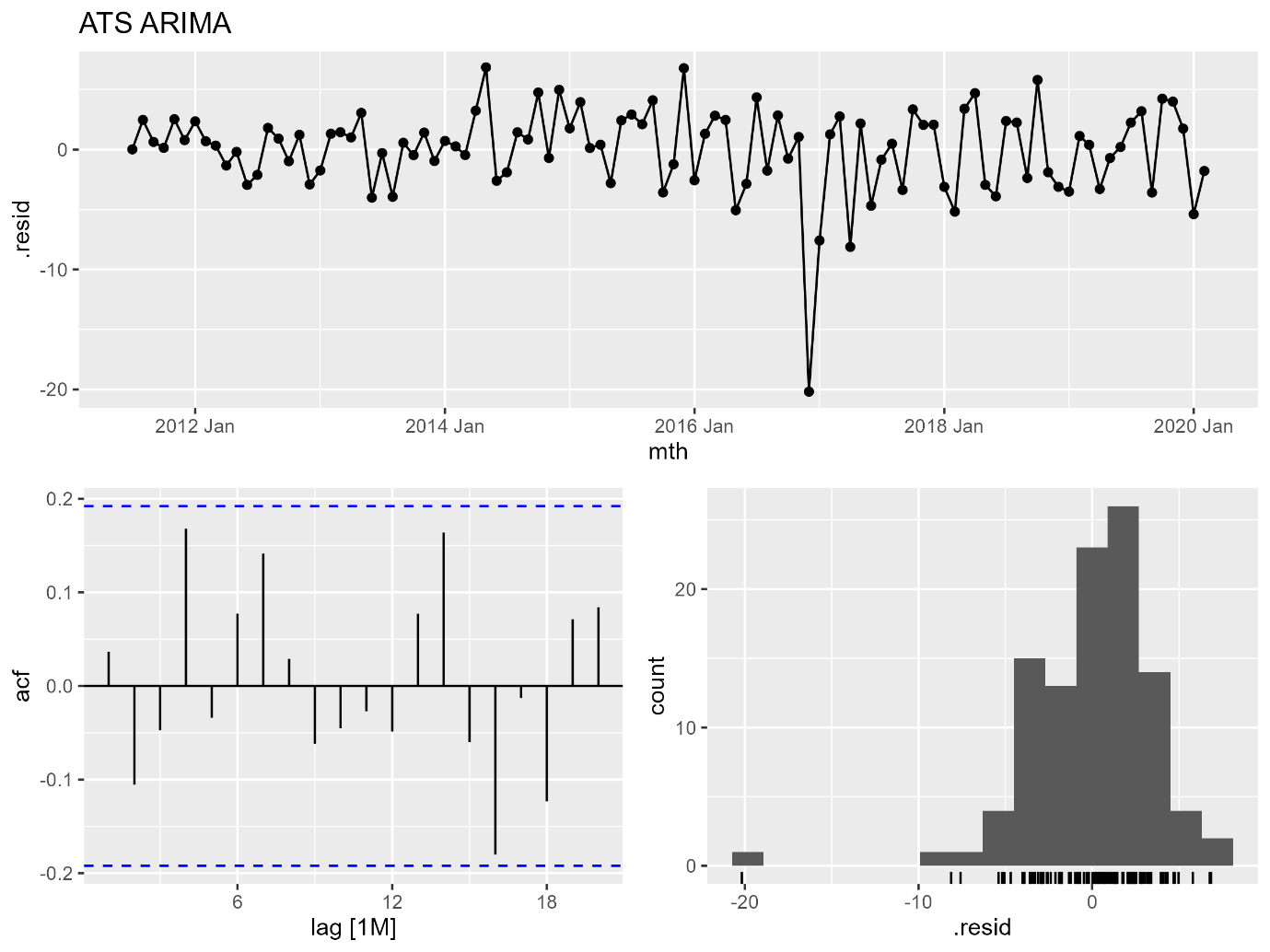


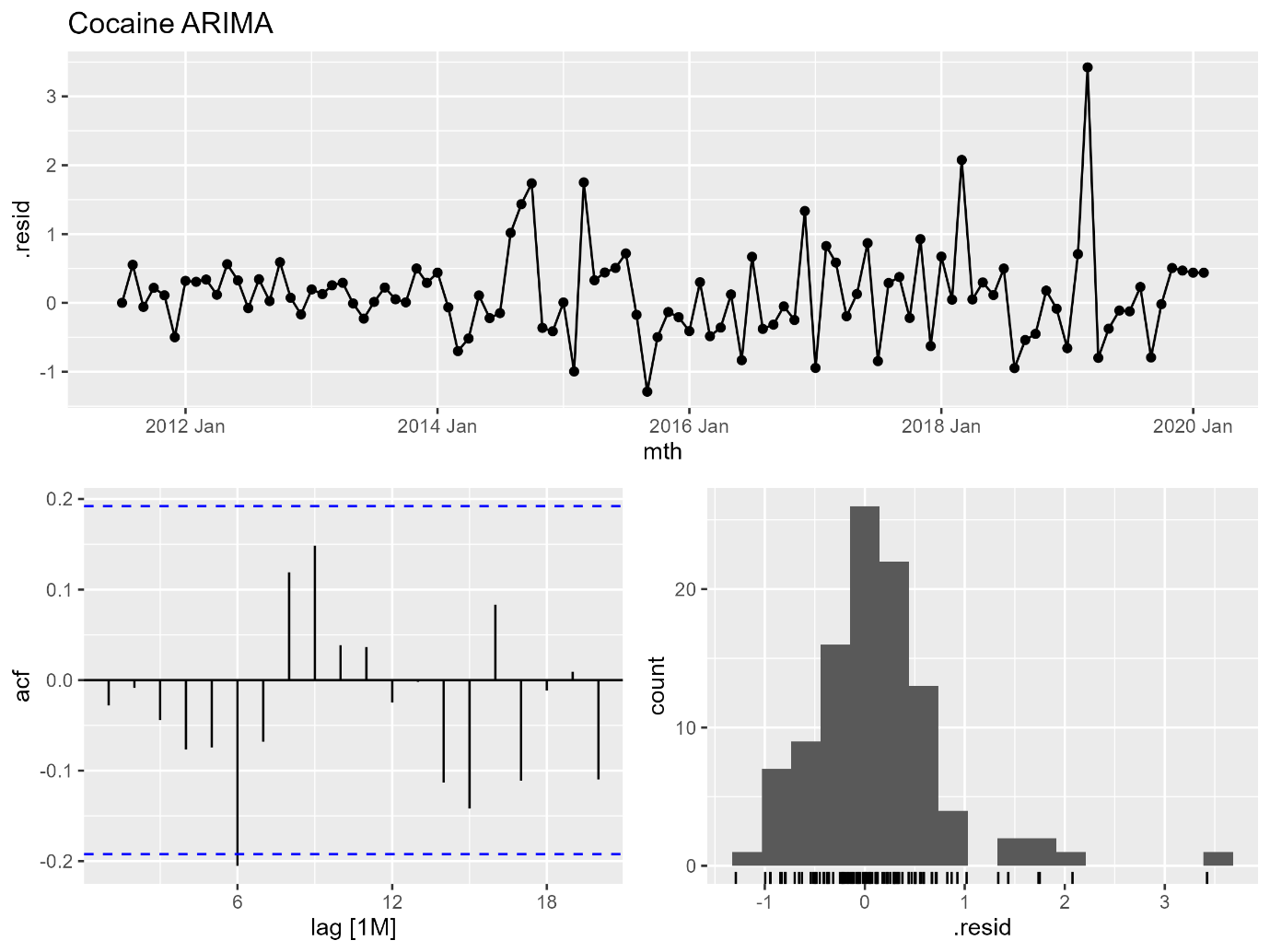


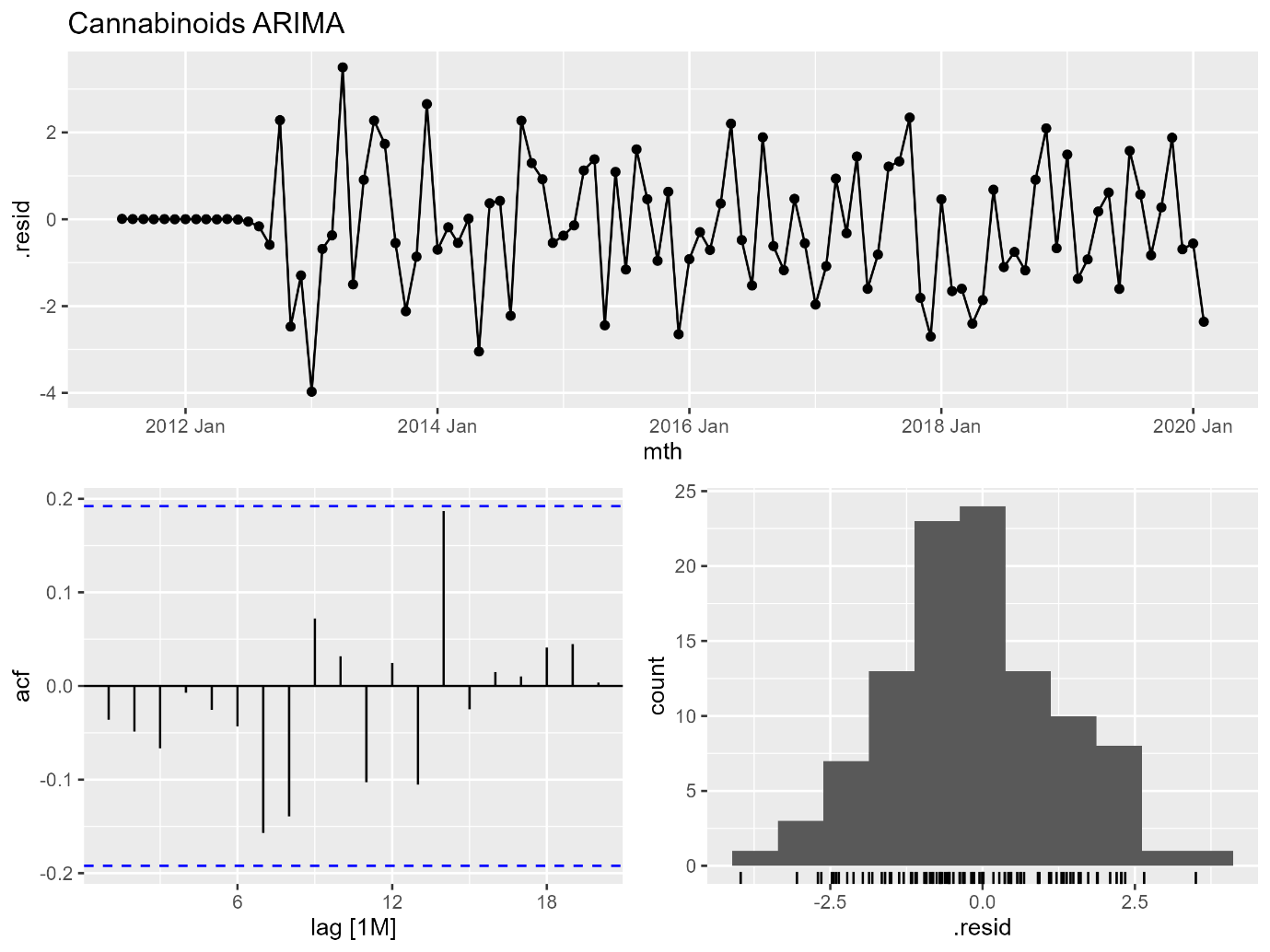


**Figure C2. Residual plots for drug-induced deaths. ARIMA, autoregressive integrated moving average; ATS, amphetamine-type stimulants**


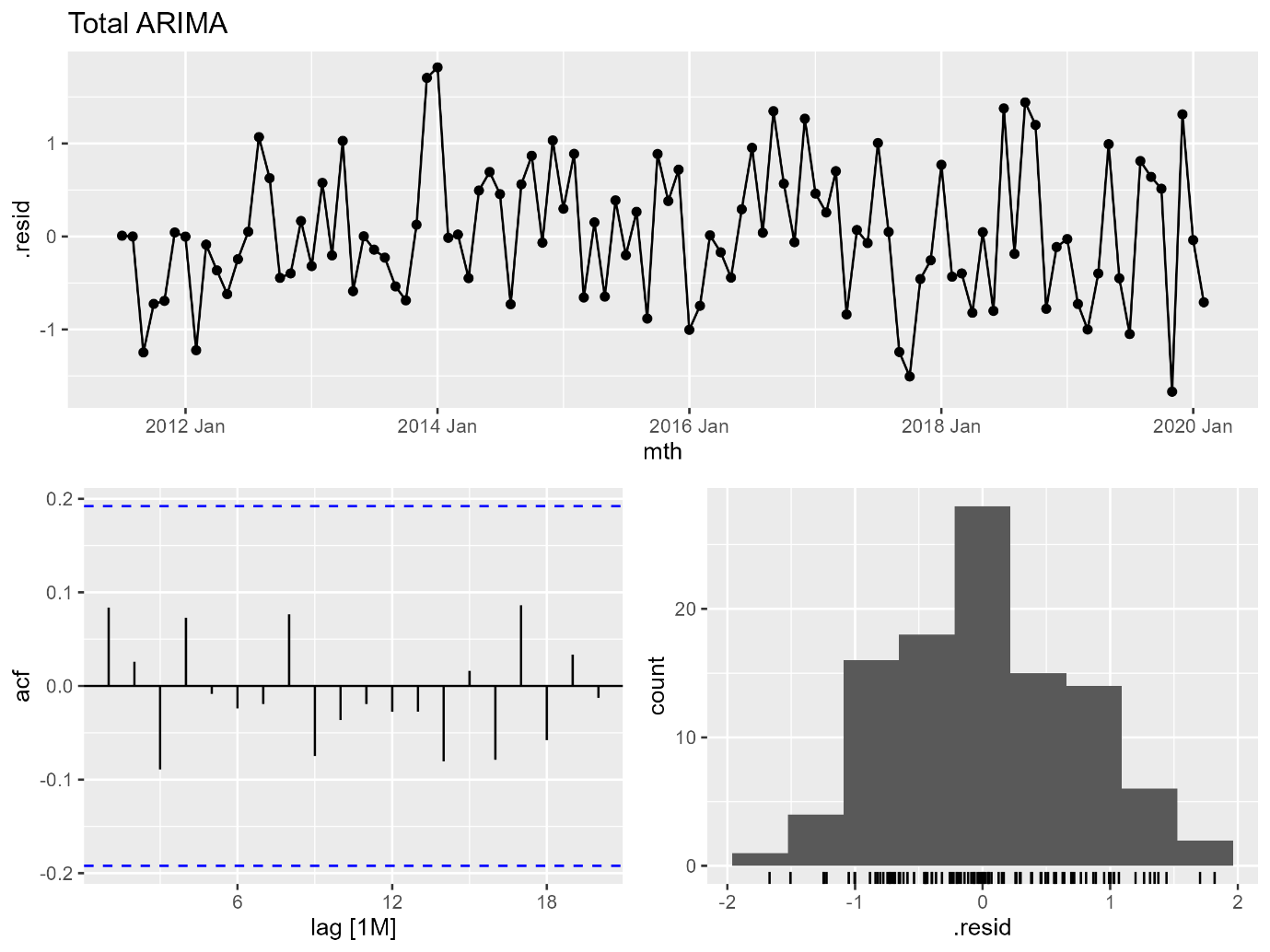


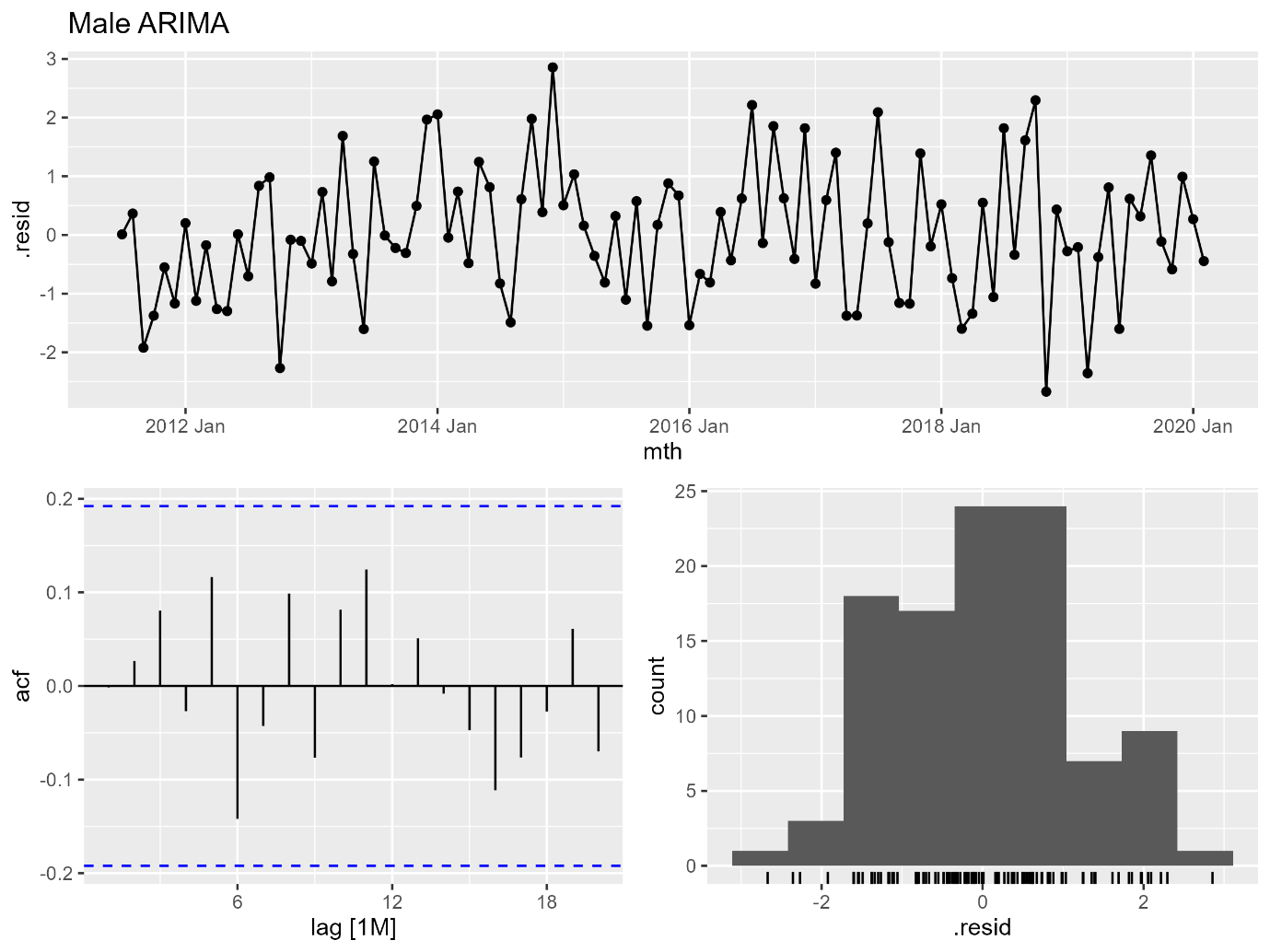


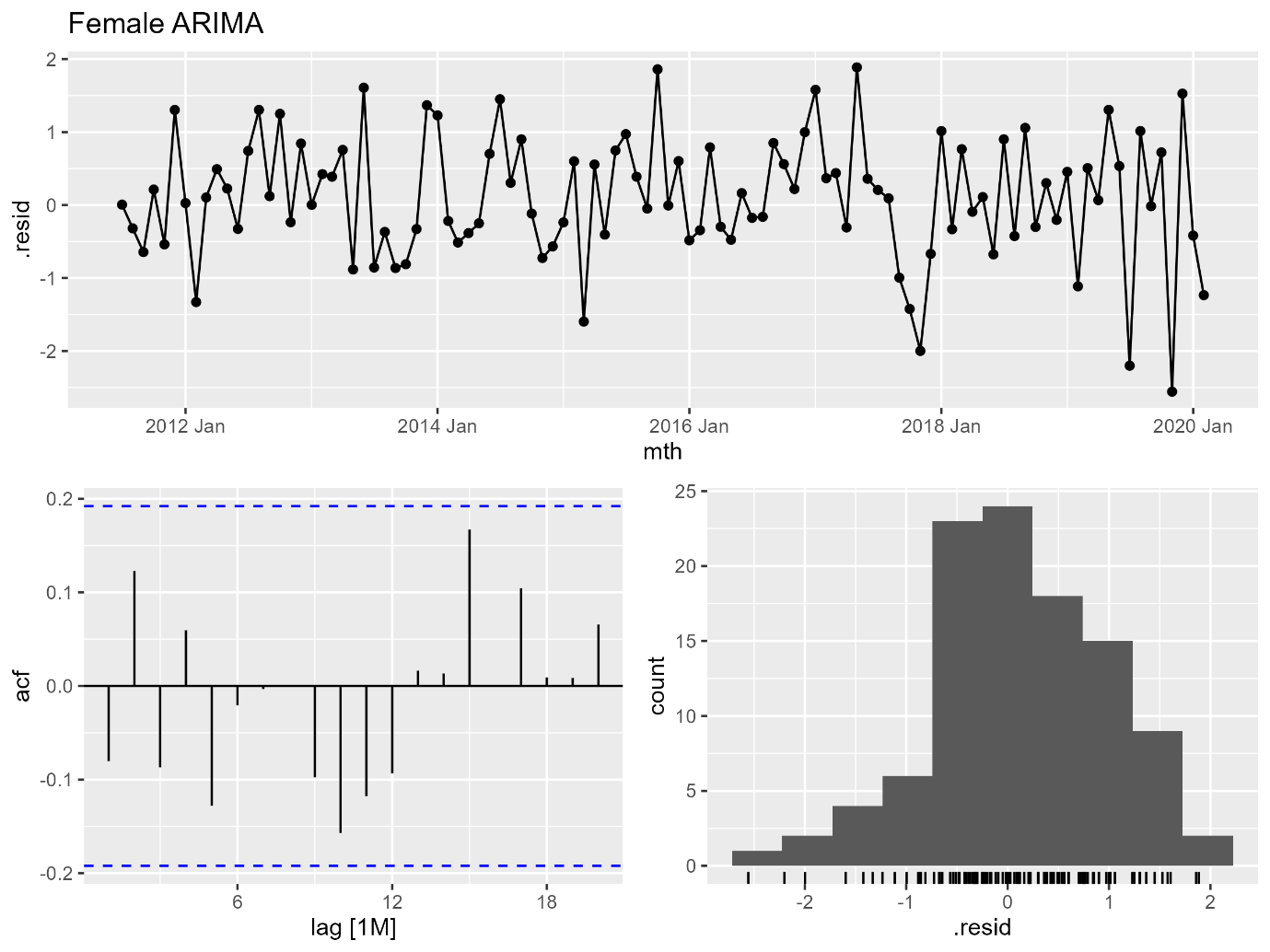


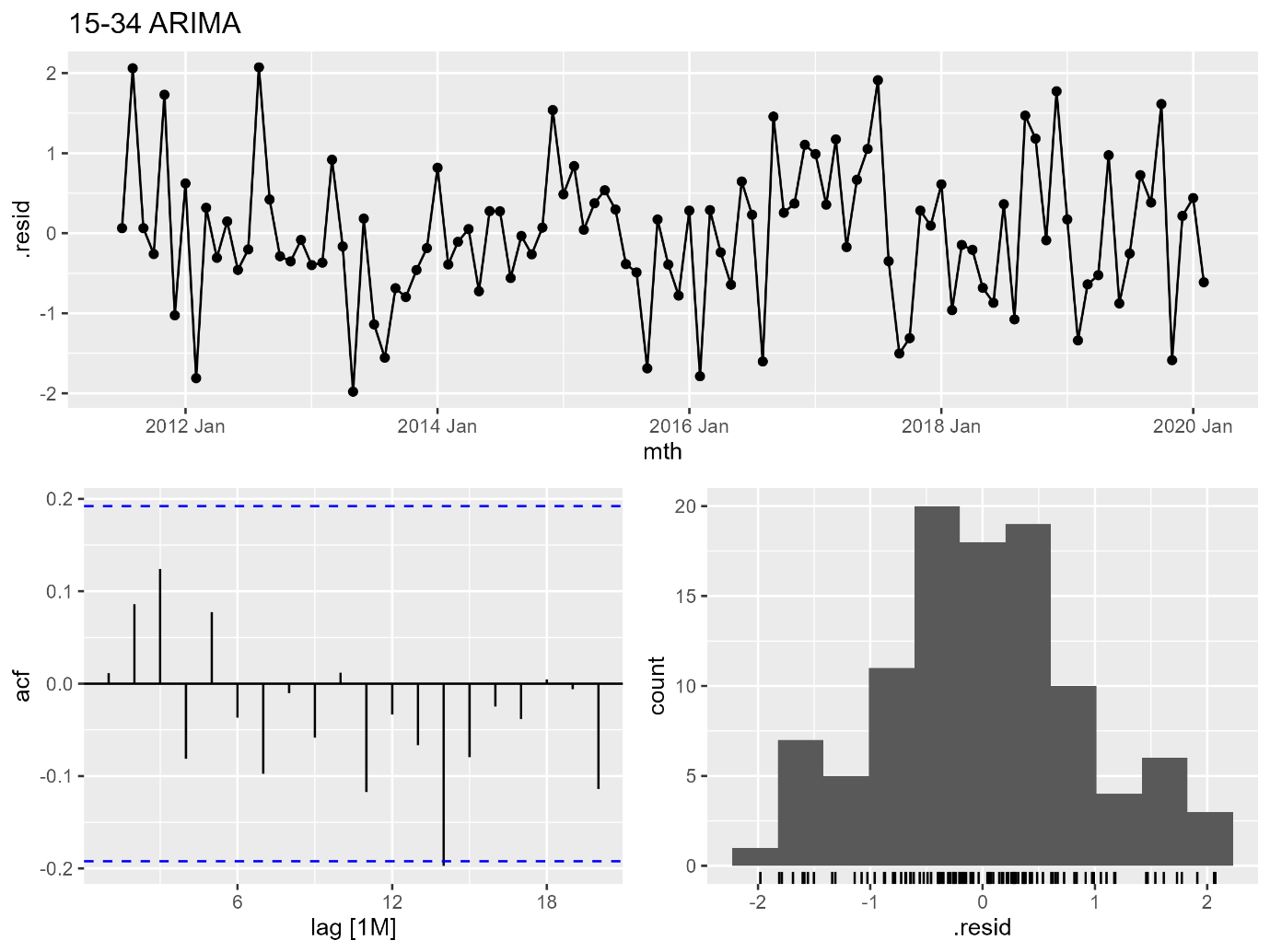


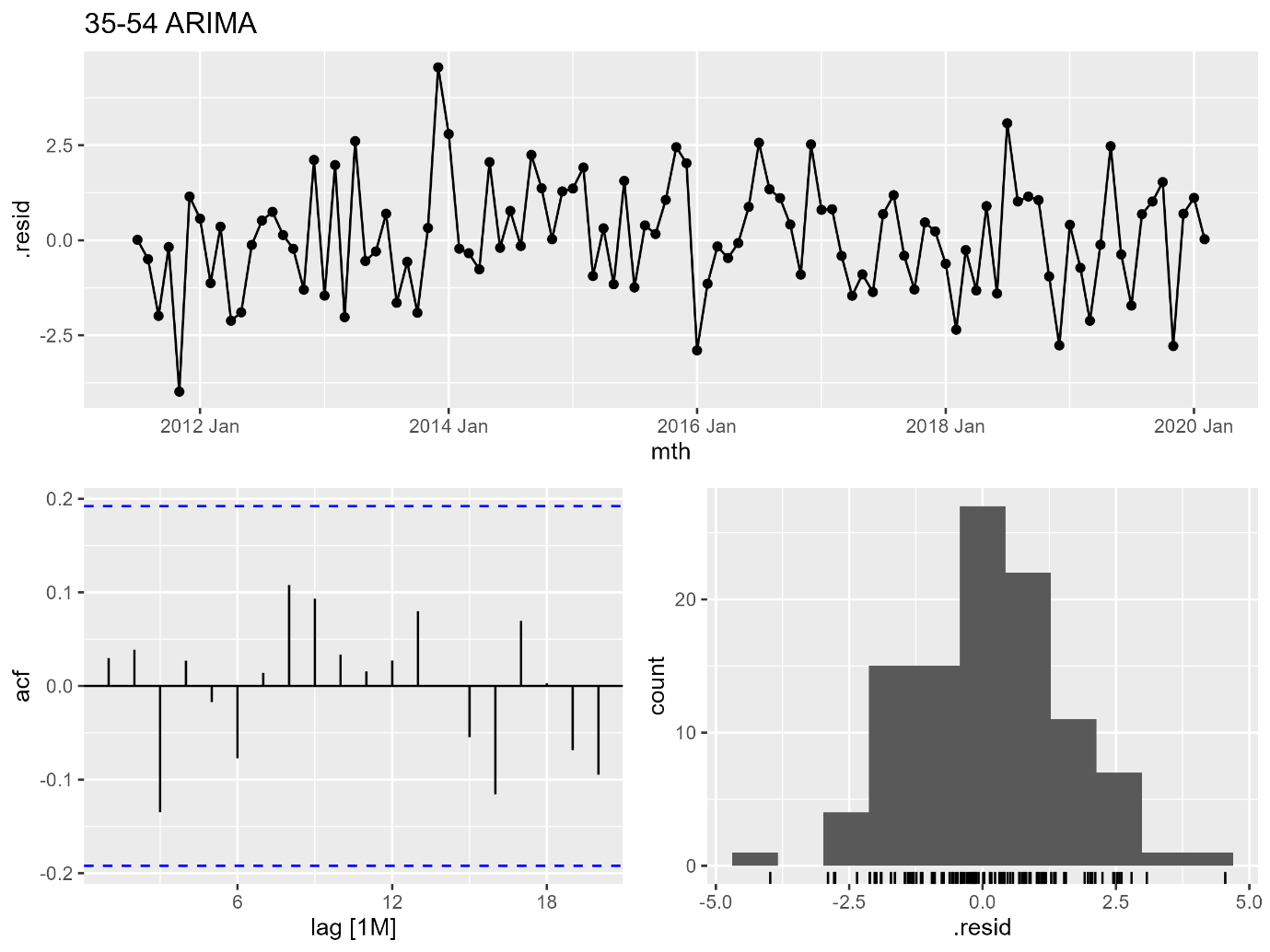


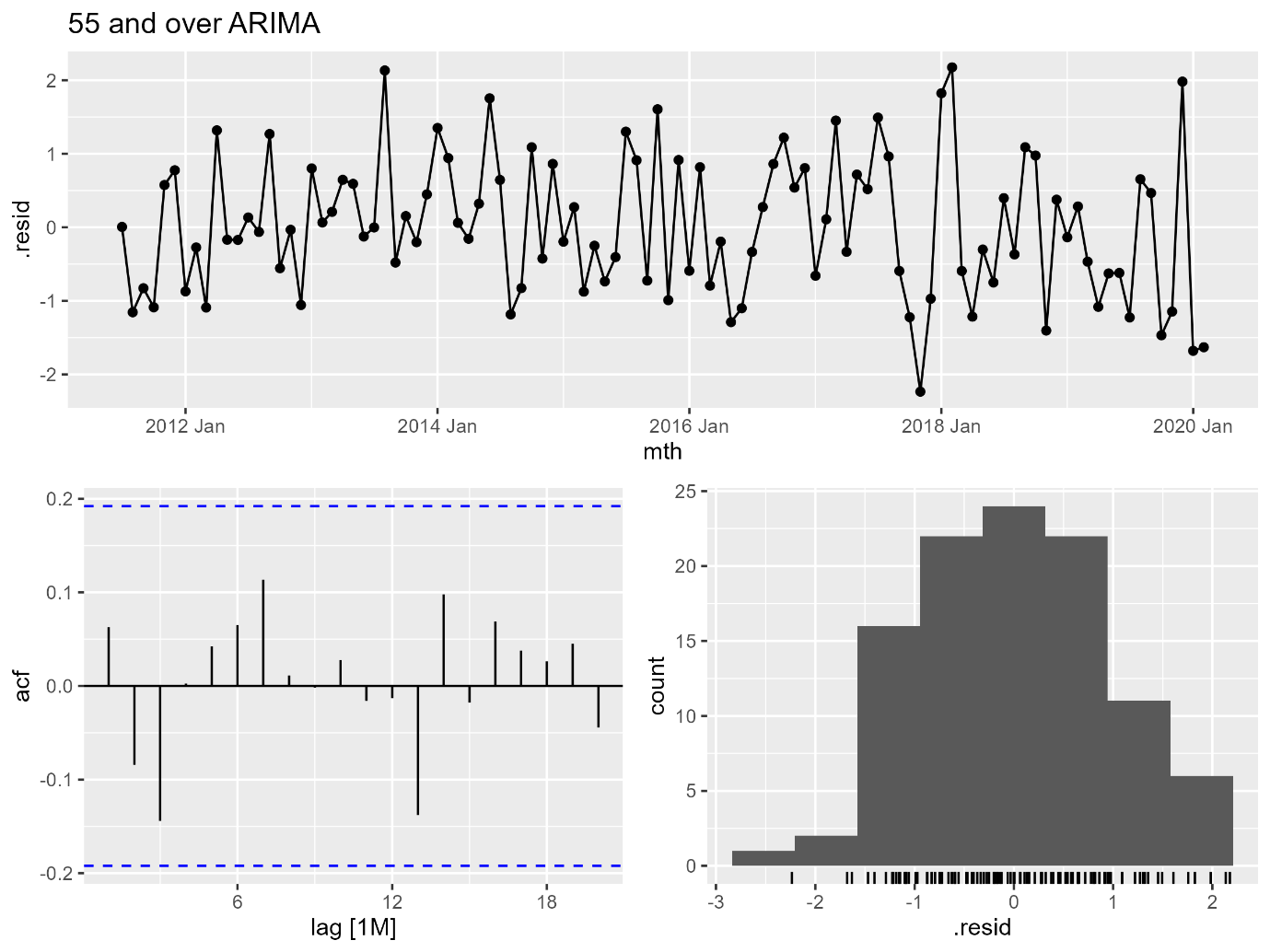


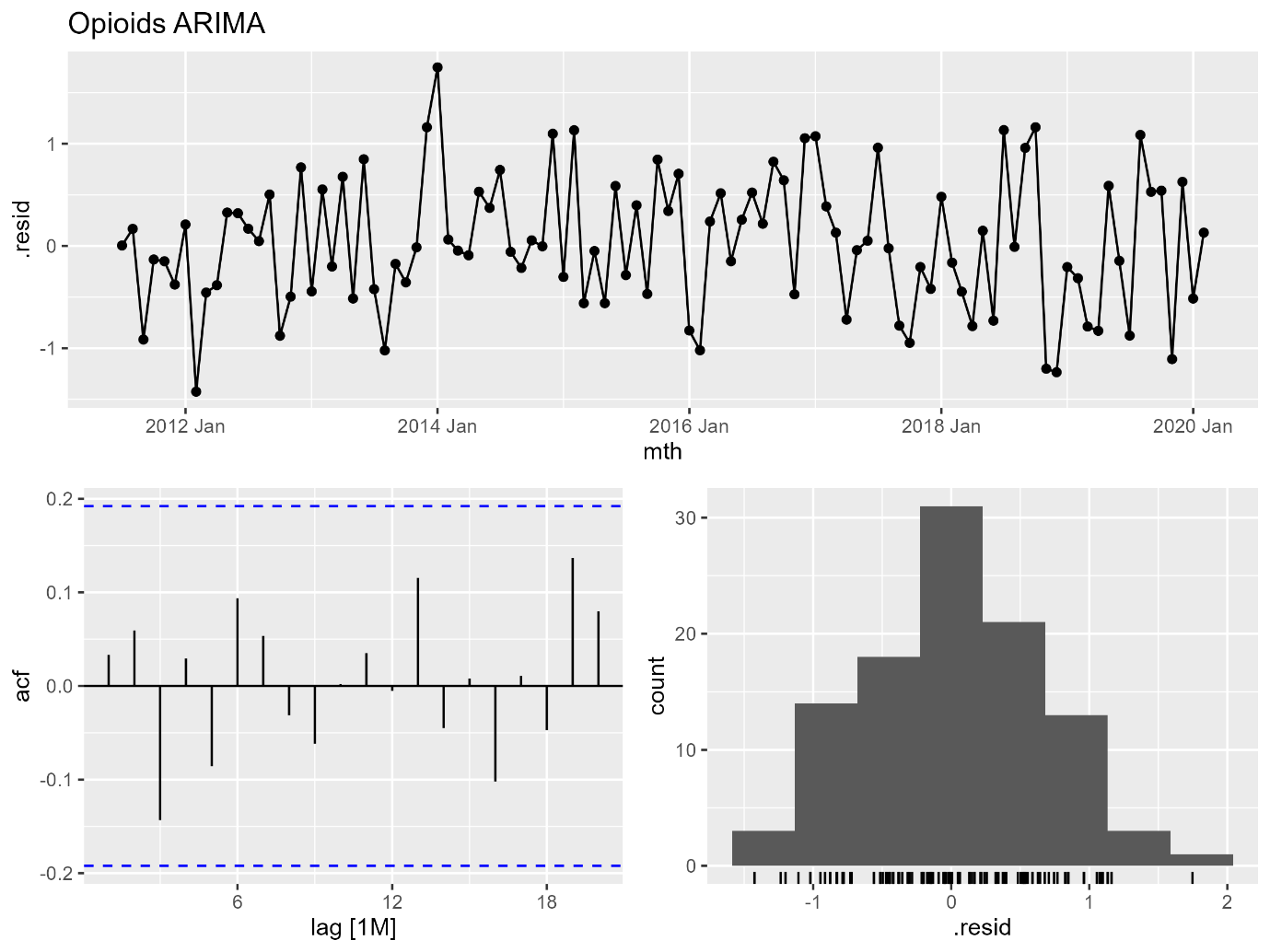


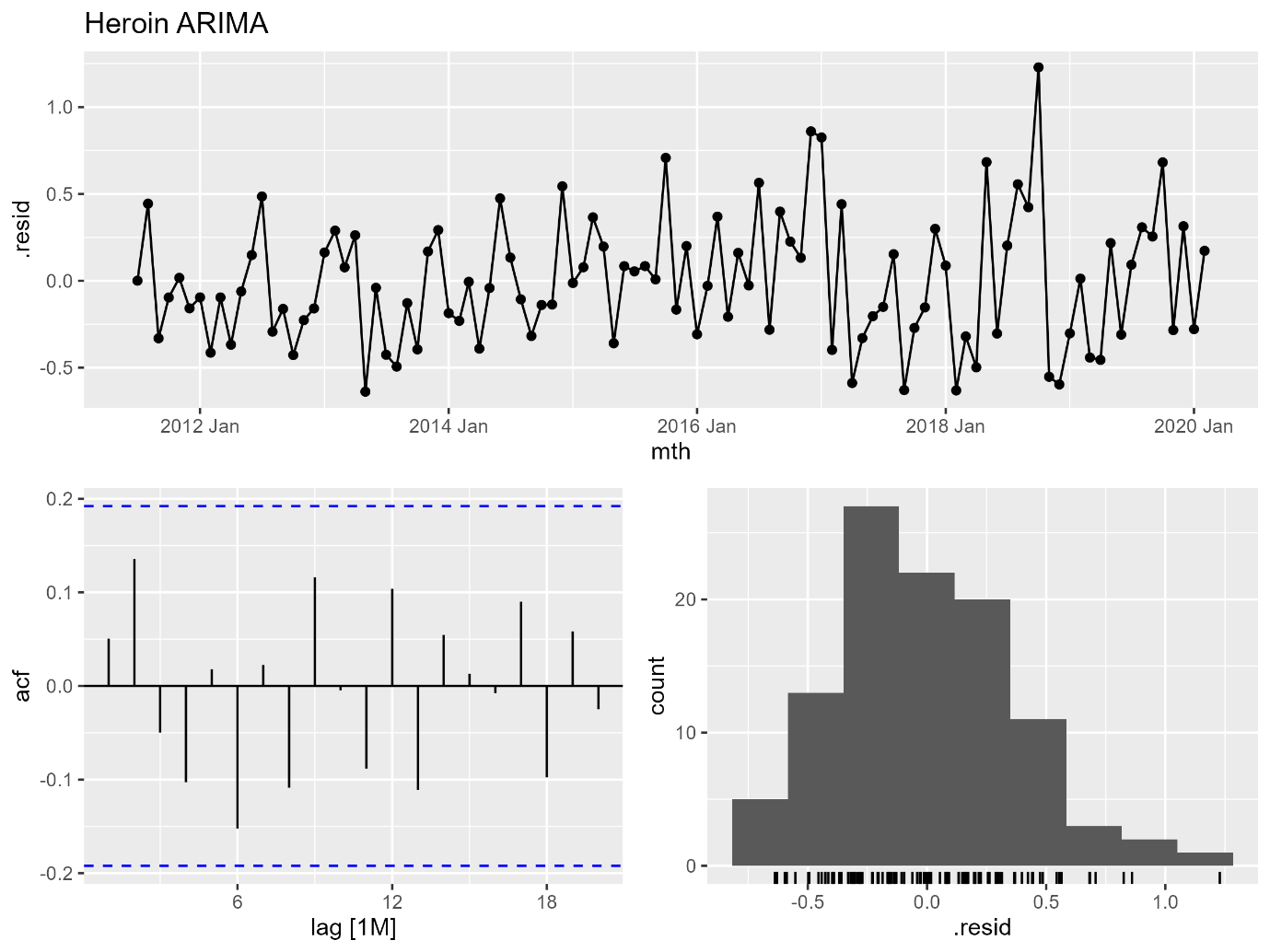


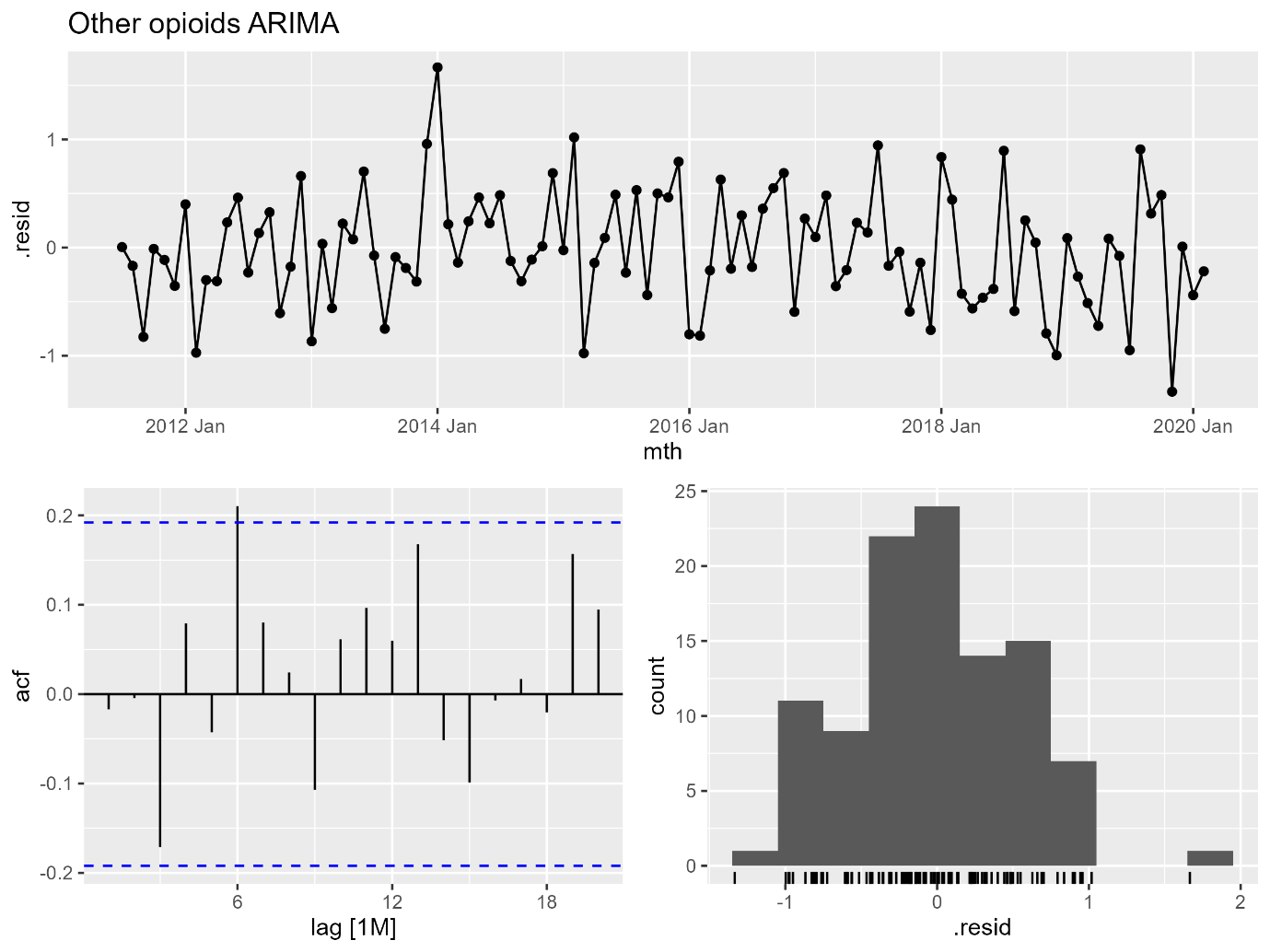


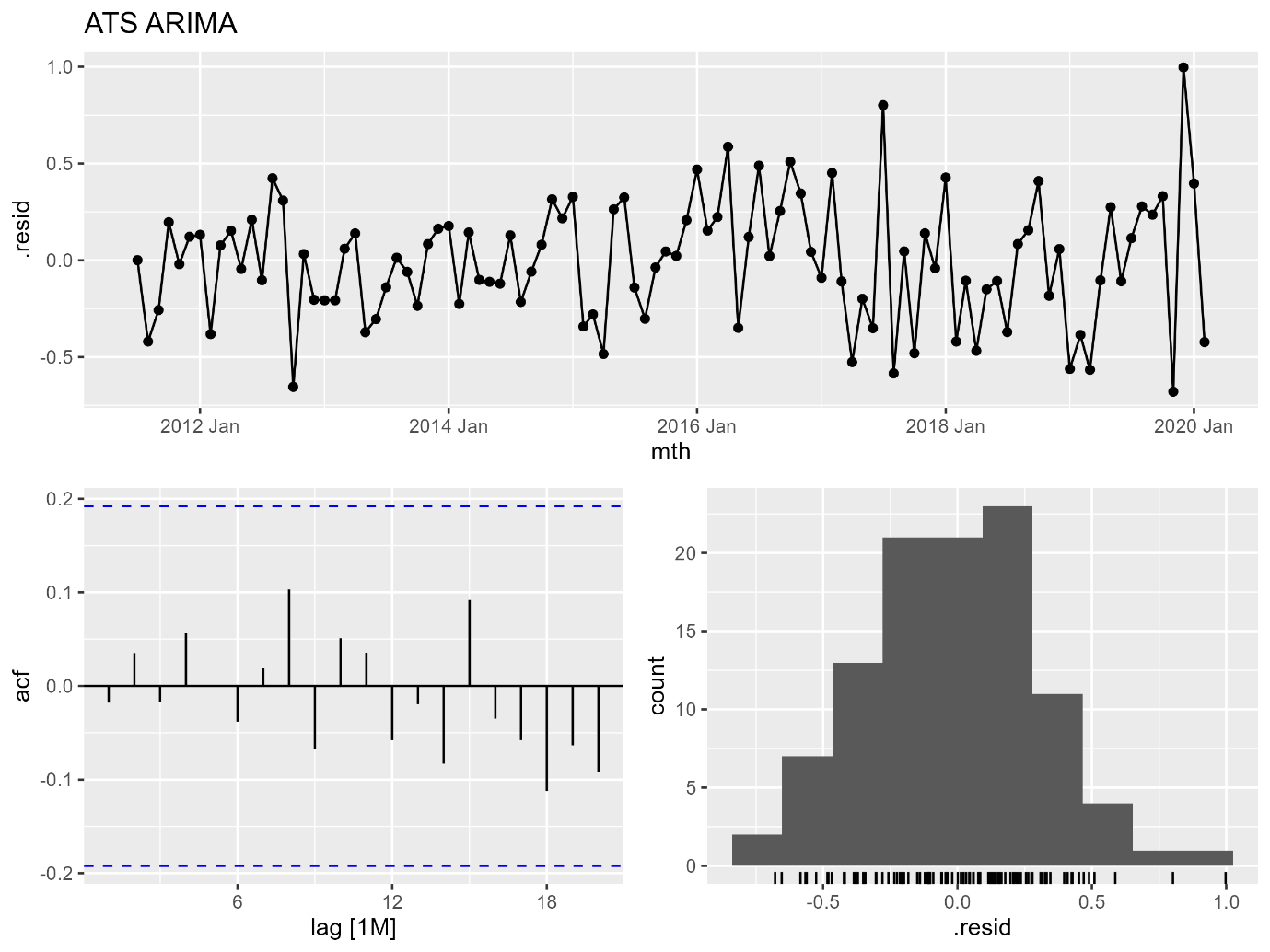


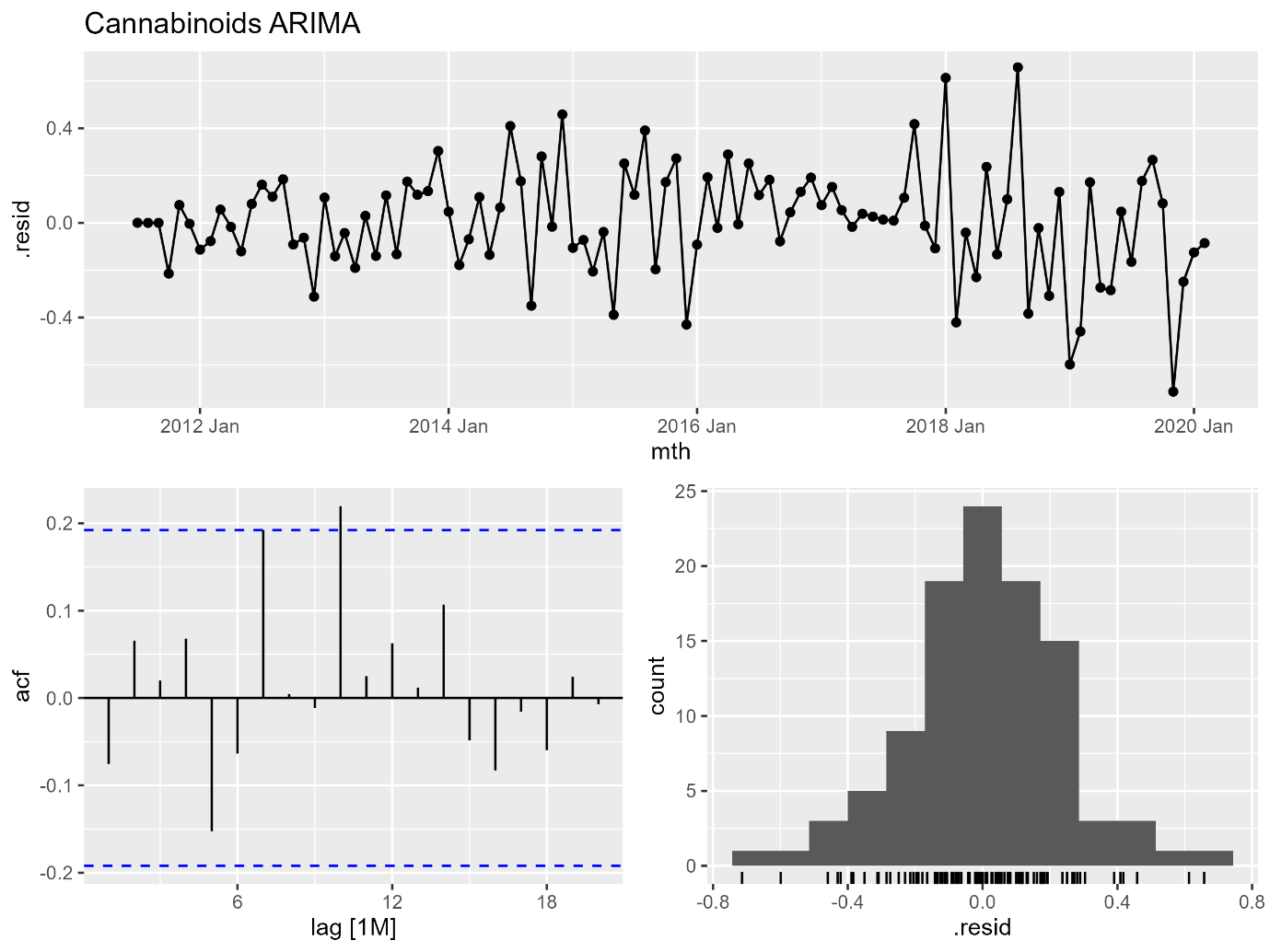


# Appendix D. Results using the shorter time series from 1 July 2016 to 30 April 2021, with and without adjusting for all-cause hospitalisation rates

**Table D1. Deviation in observed rate (per 1,000,000 persons per month) of drug-induced hospitalisations from the counterfactual forecast in the COVID-19 pandemic period, with and without adjustment for all-cause hospitalisation rates.**

|  | **Estimated deviation in rate per month (95% PI)** | |
| --- | --- | --- |
|  | **With adjustment for all-cause hospitalisation** | **Without adjustment for all-cause hospitalisation** |
| Overall | 7.0 (-9.1, 22.5) | -1.4 (-18.0, 13.8) |
| **Sex** |  |  |
| Males | -7.9 (-36.7, 22.3) | -10.3 (-32.2, 14.0) |
| Females | **22.9 (19.2, 26.7)** | **13.7 (9.2, 18.3)** |
| **Age, years** |  |  |
| 15-34 | **21.4 (1.4, 41.9)** | 16.6 (-12.7, 46.3) |
| 35-54 | -4.3 (-10.0, 2.2) | **-11.3 (-16.6, -5.9)** |
| 55+ | **-8.6 (-13.1, -4.1)** | **-8.8 (-12.7, -5.0)** |
| **Drug** |  |  |
| Opioids | **-4.1 (-7.4, -0.7)** | **-4.1 (-7.4, -0.9)** |
| Heroin | **-1.8 (-3.3, -0.5)** | **-1.8 (-3.3, -0.5)** |
| Other opioids | -1.0 (-3.4, 1.3) | -1.0 (-3.4, 1.3) |
| Amphetamine-type stimulants | -3.0 (-9.4, 4.4) | -3.3 (-9.2, 3.7) |
| Cocaine | -0.0 (-0.5, 0.4) | 0.1 (-0.2, 0.5) |
| Cannabinoids | **6.7 (6.1, 7.3)** | **4.0 (2.4, 5.5)** |

Note: 95% PI = 95% prediction interval; bolded rows indicate estimates with 95% PI that does not include 0. ‘Other opioids’ exclude opium and heroin. For data exclusions, please refer to the methods section and Appendix B (tables B1 and B3).

**Figure D1. Crude rate (per month per 1,000,000 persons) of drug-induced hospitalisations and counterfactual forecast with (A) and without (B) adjustment for all-cause hospitalisation rates, overall and by sex.**

A1 – Overall with adjustment B1 – Overall without adjustment


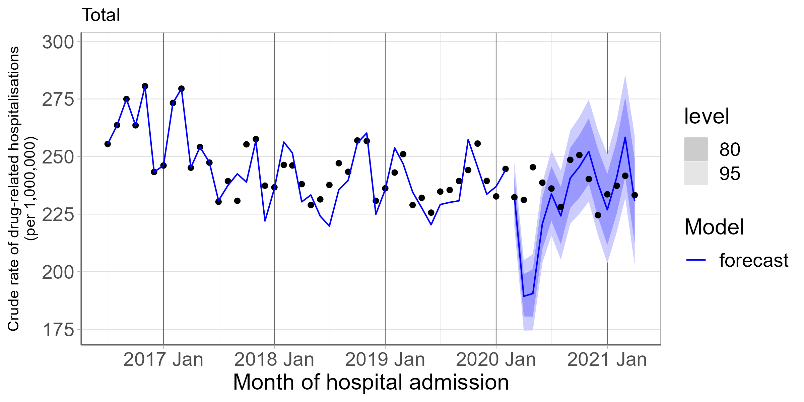

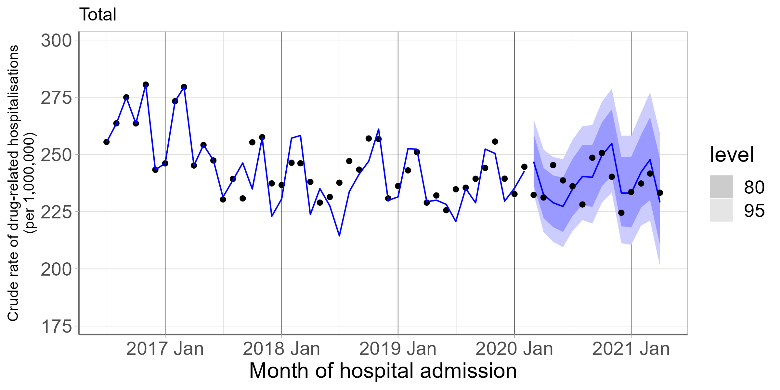


A2 – Males with adjustment B2 – Males without adjustment


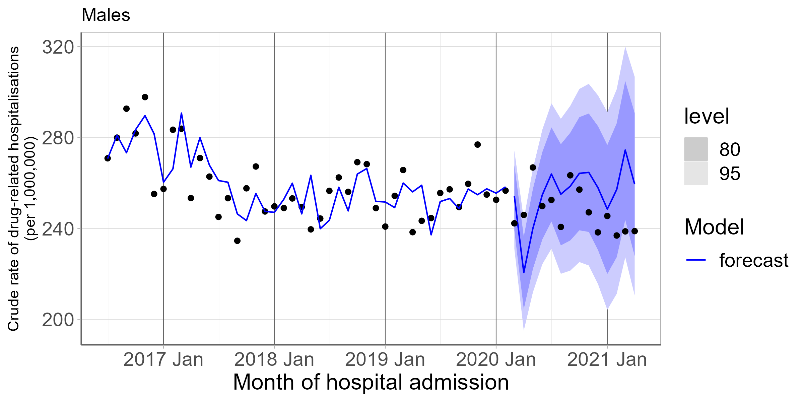

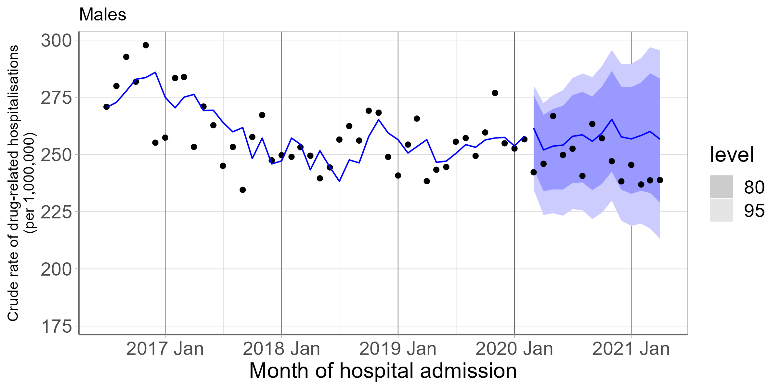


A3 – Females with adjustment B3 – Females without adjustment


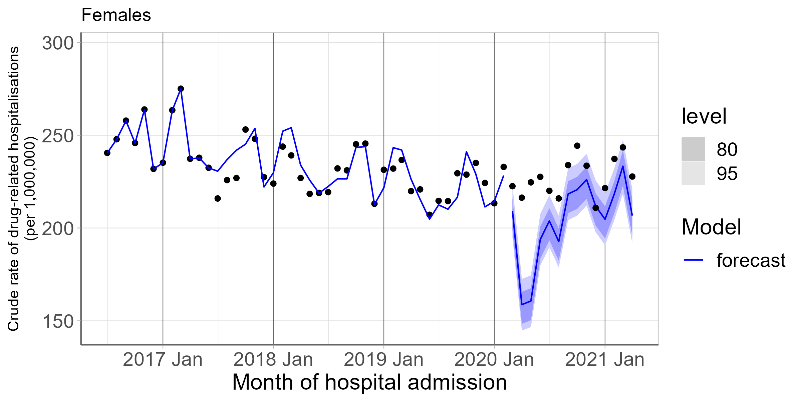

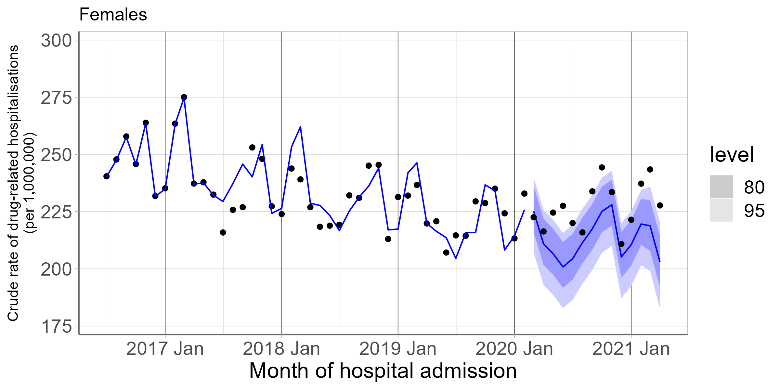


**Figure D2. Crude rate (per month per 1,000,000 persons) of drug-induced hospitalisations and counterfactual forecast with (A) and without (B) adjustment for all-cause hospitalisation rates by age.**

A1 – 15-34 years with adjustment B1 – 15-34 years without adjustment


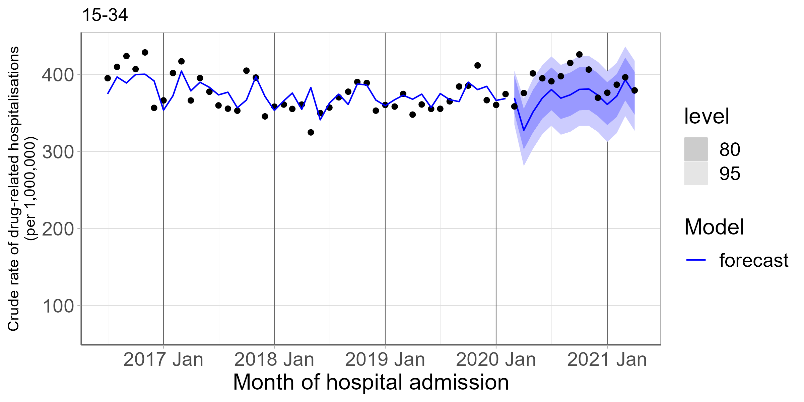

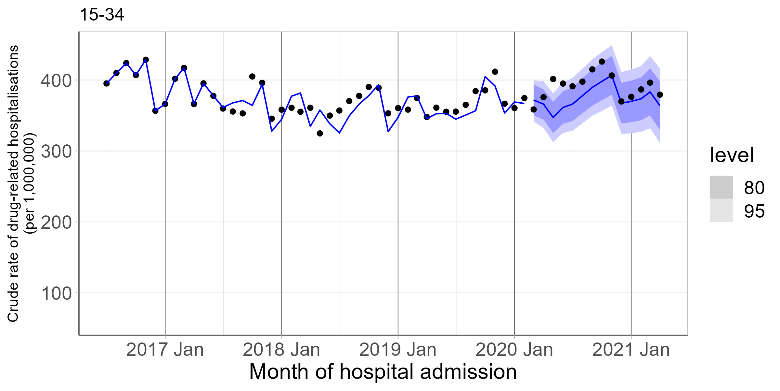


A2 – 35-54 years with adjustment B2 – 35-54 years without adjustment


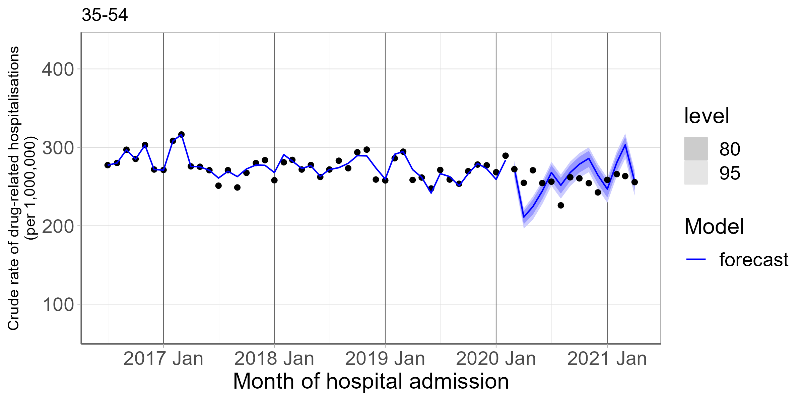

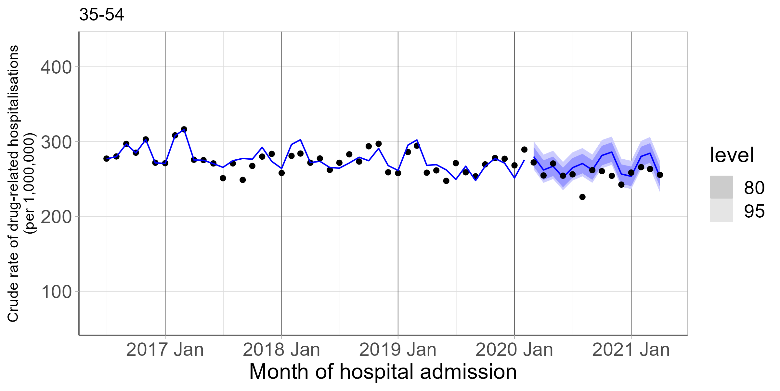


A3 – 55+ years with adjustment B3 – 55+ years without adjustment


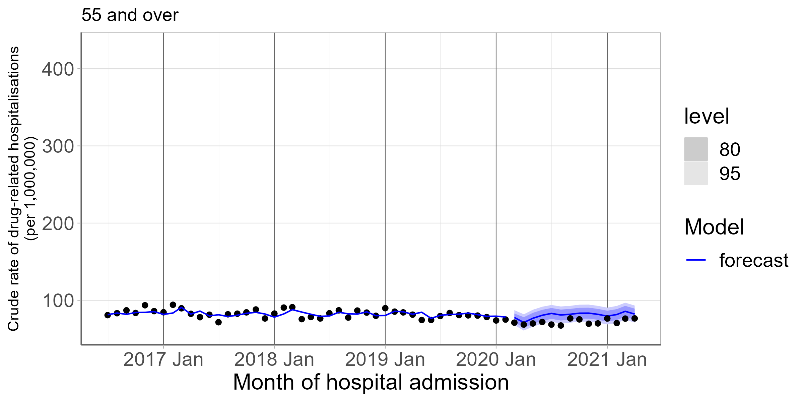

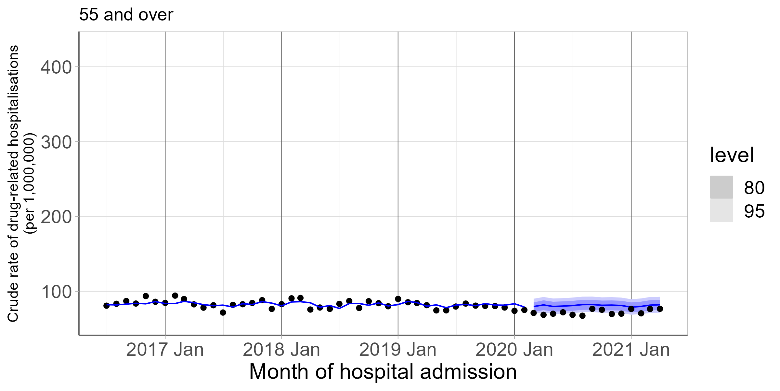


**Figure D3. Crude rate (per month per 1,000,000 persons) of opioid-induced hospitalisations and counterfactual forecast with (A) and without adjustment (B) for all-cause hospitalisation rates.**

A1 – All opioids with adjustment B1 – All opioids without adjustment


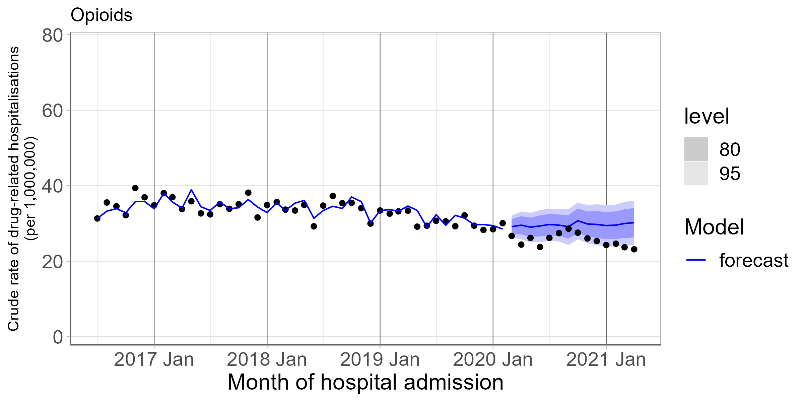

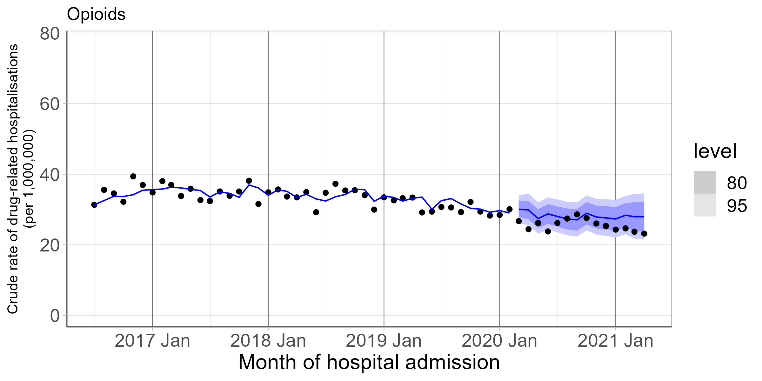


A2 – Heroin with adjustment B2 – Heroin without adjustment


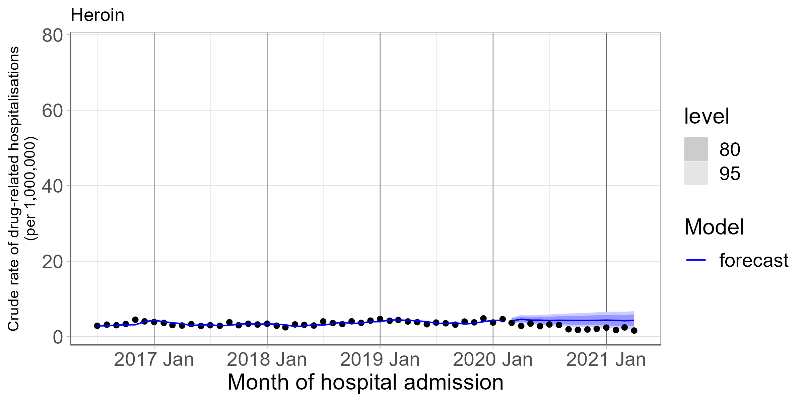

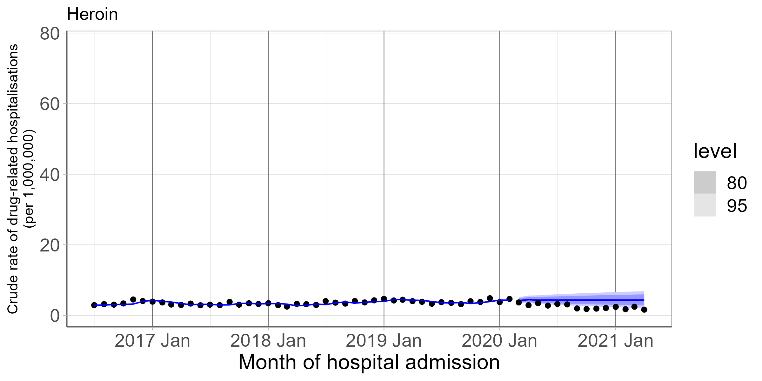


A3 – Other opioids with adjustment B3 – Other opioids without adjustment

**
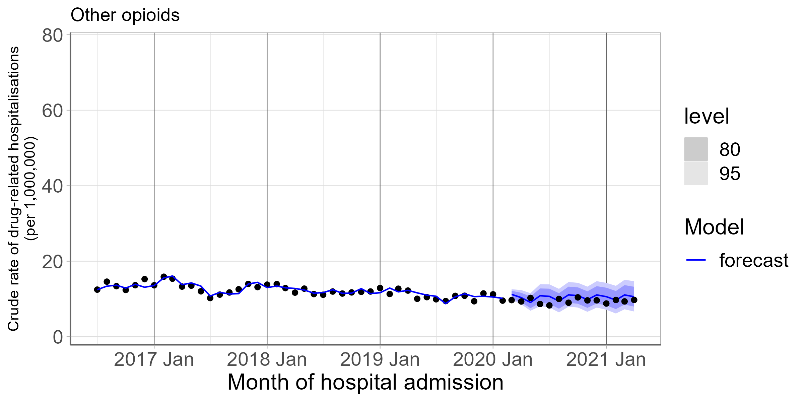

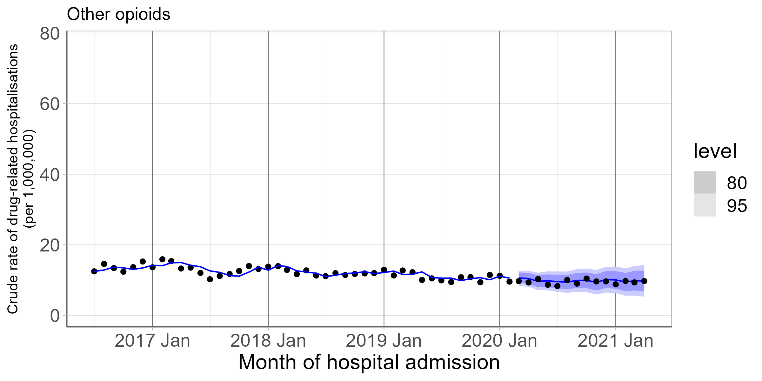
**

**Figure D4. Crude rate (per 1,000,000 persons per month) of drug-induced hospitalisations involving amphetamine-type stimulants (ATS), cocaine and cannabinoids and counterfactual forecast with and without adjustment for all-cause hospitalisation rates.**

A1 – ATS with adjustment B1 – ATS without adjustment


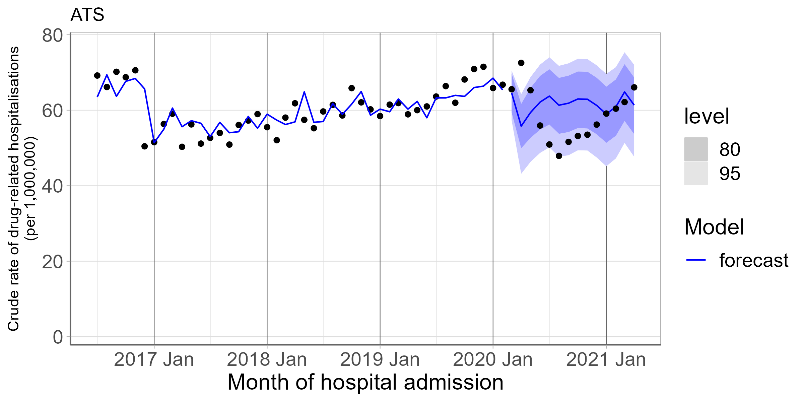

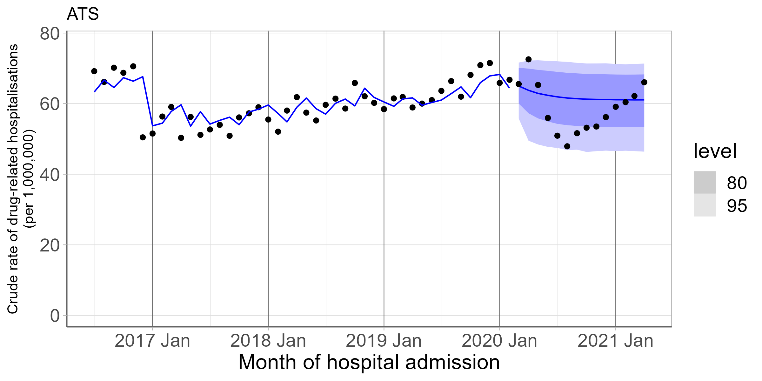


A2 – Cocaine with adjustment B2 – Cocaine without adjustment


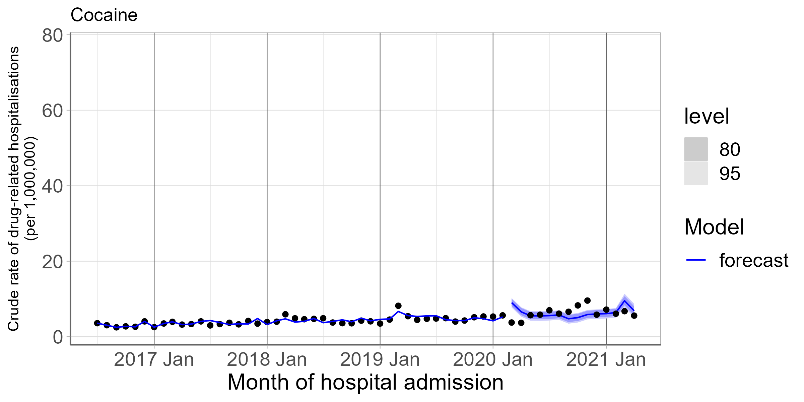

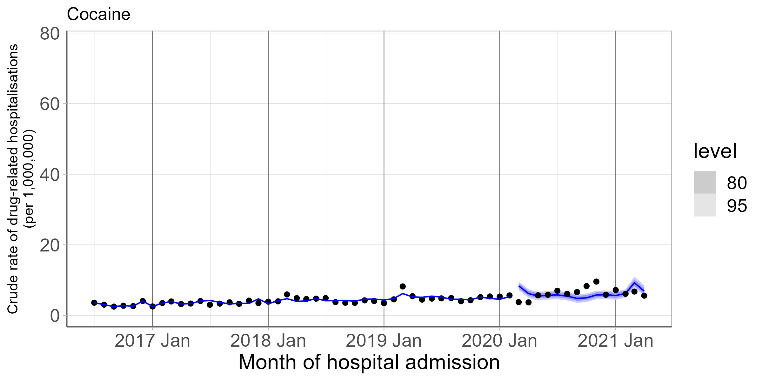


A3 – Cannabinoids with adjustment B3 – Cannabinoids without adjustment


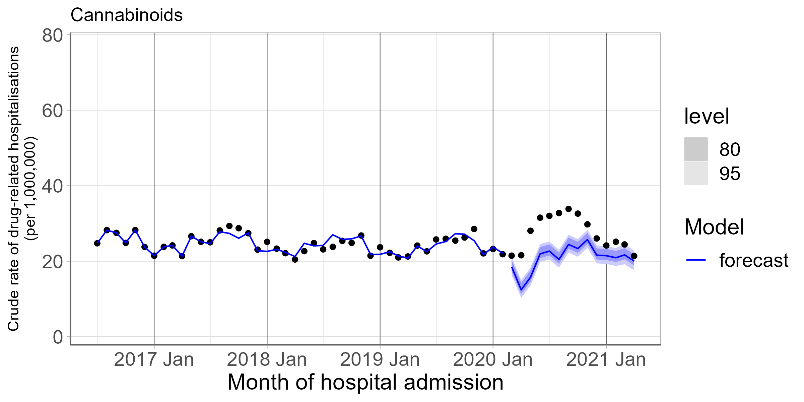

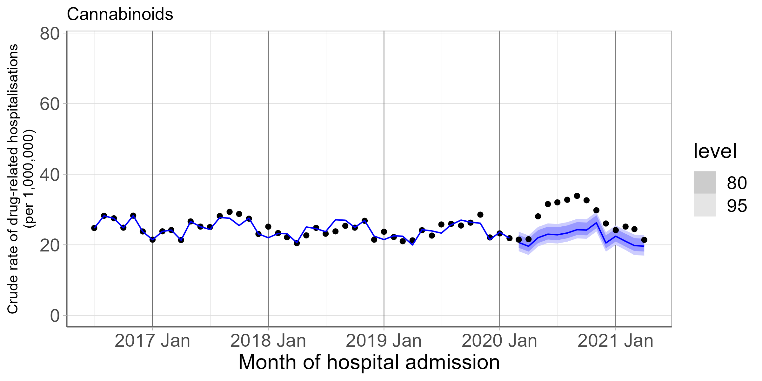

Supplement: Supplementary file 1 — Data S1. [file DAR-44-1419-s001.docx]
